# Identification of Isoflavonoid Biosynthesis-Related R2R3-MYB Transcription Factors in *Callerya speciosa* (Champ. ex Benth.) Schot Using Transcriptome-Based Gene Coexpression Analysis

**DOI:** 10.1155/2021/9939403

**Published:** 2021-05-25

**Authors:** Linchan Yu, Ding Huang, Jinyuan Gu, Dongjin Pan, Yong Tan, Rongshao Huang, Shaochang Yao

**Affiliations:** ^1^College of Pharmacy, Guangxi University of Chinese Medicine, Nanning, Guangxi, China; ^2^Institute of Marine Drugs, Guangxi University of Chinese Medicine, Nanning, Guangxi, China

## Abstract

The R2R3-MYB family is one of the largest plant transcription factor (TF) families playing vital roles in defense, plant growth, and secondary metabolism biosynthesis. Although this gene family has been studied in many species, isoflavonoid biosynthesis-related R2R3-MYB TFs in *Callerya speciosa* (Champ. ex Benth.) Schot, a traditional Chinese medicinal herb, are poorly understood. Here, a total of 101 R2R3-MYB TFs were identified from *C. speciosa* transcriptome dataset. 25 clades divided into five functional groups were clustered based on the sequence similarity and phylogenetic tree. Conserved motifs and domain distribution, expression patterns, and coexpression networks were also employed to identify the potential R2R3-MYB TFs in the regulation of isoflavonoid biosynthesis. *In silico* evaluation showed that the deduced R2R3-CsMYB proteins contain highly conserved R2R3 repeat domain at the N-terminal region, that is the signature motif of R2R3-type MYB TFs. Eight potential TFs (*CsMYB17*, *CsMYB36*, *CsMYB41*, *CsMYB44*, *CsMYB45*, *CsMYB46*, *CsMYB72*, and *CsMYB81*) had high degrees of coexpression with four key isoflavonoid biosynthetic genes (*CsIFS*, *CsCHS7*, *CsHID-1*, and *CsCHI3*), in which *CsMYB36* as a potential regulator possessed the highest degree. HPLC analysis showed that formononetin and maackiain contents were significantly increased during the development of tuberous roots, which might be controlled by both related R2R3-CsMYBs and structural genes involved in the isoflavonoid biosynthesis pathway. The transcriptome data were further validated by reverse transcription real-time PCR (RT-qPCR) analysis, and similar expression profiles between TFs and key structural genes were identified. This study was the first step toward the understanding of the R2R3-MYB TFs regulating isoflavonoid biosynthesis in *C. speciosa*. The results will provide information for further functional analysis and quality improvement through genetic manipulation of these potential R2R3-CsMYB genes in *C. speciosa*.

## 1. Introduction

*Callerya speciosa* (Champ. ex Benth.) Schot, a medicinal plant of the *Fabaceae* family, has a long cultivation history in south China and is traditionally used as a medicine for enriching weakness, as well as strengthening bones and muscles. Modern pharmacological research has shown that *C. speciosa* can strengthen immunity, nourish lungs, exhibit anti-inflammatory and anti-asthmatic effects [1]. Isoflavonoids are specialized metabolites produced from the tuberous roots of *C. speciosa*, including the index compounds maackiain and formononetin [2, 3].

Isoflavonoids are reported to be regulated by several structural genes in the (iso) flavonoid biosynthetic pathway, and those structural genes isolated and characterized to date are conserved in higher plants [4, 5]. The isoflavonoid C6–C3–C6 carbon skeletons are derived from *p*-coumaroyl-CoA, which is produced with the coordinated reaction of three key enzymes in the phenylpropanoid pathway: phenylalanine ammonialyase (PAL), cinnamate 4-hydroxylase (C4H), and 4-coumarate-CoA ligase (4CL). Chalcone synthase (CHS) can catalyze *p*-coumaroyl-CoA to naringenin chalcone alone, or it functions with chalcone reductase (CHR) to synthesize isoliquiritigenin chalcone. The products are further catalyzed by chalcone isomerase (CHI) to yield liquiritigenin [6]. Then, some of the downstream key enzymes of the isoflavonoid pathway are 2-hydroxyisoflavanone dehydratase (HID), isoflavone synthase (IFS), isoflavone 4′-O-methyltransferase (HI4′OMT), and vestitone reductase (VR), under the reactions of hydroxylation, dehydration, methylation, and glycosylation to form various isoflavonoid derivatives [7]. Recently, the coordinated expression of structural genes in the (iso) flavonoid biosynthetic pathway has been analyzed via the transcriptome data in several legume plant species, such as soybean, *Pueraria candollei*, and *Pueraria lobata* [6, 8, 9]. In *C. speciosa*, we have previously reported that 15 key genes might be involved in the isoflavonoid biosynthesis pathway, including *IFSs*, *CHSs*, and *VRs* [10].

In general, metabolite accumulation can be better controlled by coordinated regulation of key regulatory genes (i.e., TFs) in multiple steps, rather than modifying a single structural gene [11, 12]. More potential TFs and their target genes need to be characterized. The myeloblastosis (MYB) TFs are widespread in plants and are characterized by a conserved MYB DNA-binding domain at the N-terminus, which form a helix-turn-helix (HTH) structure of approximately 53 amino acid residues [13]. The R2R3-MYB IFs, which are the largest subfamily of MYB family TFs, contain two imperfect repeats in the MYB DNA-binding domain. They act by directly binding to the promoters of structural genes in the biosynthetic pathway or generally forming a ternary complex with basic helix-loop-helix (bHLH) and WD-repeat proteins [14]. They were reported to play essential roles in secondary metabolism, hormonal signaling, and response to stress during plant growth and development [15]. For example, several R2R3-MYB IFs were identified to regulate the biosynthesis of flavonoids, including anthocyanins, flavonol, proanthocyanidins, and isoflavonoids. AtMYB11 and AtMYB12 positively regulated the biosynthesis of flavonol [16], while AtMYB111 played a vital role in proanthocyanidin biosynthesis in *Arabidopsis* [17]. Several R2R3-MYB proteins (GmMYB29, GmMYB12B2, GmMYB176, GmMYB133, GmMYB58, and GmMYB205) in *Glycine max* were identified as activators of isoflavonoid biosynthesis by up-regulating the expression of key structural genes [11, 12, 18–20]. *LjMYB14*, which encoded R2R3-MYB protein in *L. japonicas*, positively modulated the structural genes in the phenylpropanoid and isoflavonoid biosynthetic pathways [21]. PmMYB18 and PmMYB75 might be positive regulators of isoflavonoid biosynthesis in *P. candollei* var. mirifica [9]. GbMYBFL was involved in flavonoid biosynthesis, which played a critical role in flavonoids and anthocyanin accumulation in *Ginkgo biloba* [22]. Considering the vital roles of R2R3-MYB TFs in secondary metabolism, identification and characterization of the R2R3-MYB TFs in *C. speciosa* can help further research on the regulation of isoflavonoid biosynthesis. To date, none of R2R3-MYB TFs has been genetically studied for their involvement in regulating isoflavonoid biosynthesis in *C. speciosa*.

In the present study, a transcriptome-wide identification of R2R3-MYB TFs in *C. speciosa* was performed using the transcriptomic data. The characteristics of homologous domains and motif composition of R2R3-MYB members were investigated. We further analyzed the coexpression relationship between isoflavonoid biosynthesis-related genes and the putative R2R3-MYB TFs, which might be involved in the regulation of isoflavonoid biosynthesis. Finally, we validated the expression patterns of potential R2R3-MYB TFs and key structural genes in the isoflavonoid biosynthesis pathway by RT-qPCR analysis. Thus, a starting point for further functional analysis of R2R3-MYB TFs was performed in this study, which can offer candidate R2R3-MYB TFs for further studies to identify the roles of these genes for isoflavonoid biosynthesis in *C. speciosa*.

## 2. Results

### 2.1. Identification of R2R3-MYB Subfamily Genes in *C. speciosa*

After removing short sequences and redundant sequences, a comprehensive comparison analysis identified 101 genes encoding R2R3-MYB proteins in the MYB subfamily from the transcriptome dataset of *C. speciosa*, named CsMYB1–CsMYB101. The length of the translated putative protein sequences ranged from 105 to 1050 amino acids. The calculated molecular weight (MW) of R2R3-CsMYBs ranged from 12.16 kDa to 116.22 kDa. The calculated theoretical isoelectric points (pIs) ranged from 4.77 to 9.77. CsMYB11 possessed the longest sequence with 1050 amino acids and the largest MW of 116.22 kDa, while the shortest protein (105 amino acids) was CsMYB60 with the smallest MW of 12.16 kDa. Alpha-helix and random coil were the main secondary structures of R2R3-CsMYBs. Subcellular localization prediction results indicated that all R3R3-MYB proteins localized in the nucleus (ESM_1).

Multiple sequence alignment was also performed by *Clustal X* 2.0 to identify the characteristics of homologous domains in 101 R2R3-CsMYBs. As shown in ESM_2, the R2R3-CsMYBs contained typical R2 repeat, with basic R2 structures of [-W-(X19)-W-(X18)-W-]. A highly conserved triplet of tryptophan (W) was included in R2 repeat residues, and each tryptophan was separated by 18 or 19 amino acids. Besides the highly conserved W, several conserved positions were also identified in the R2 repeat, such as glutamic (E), aspartic (D), and arginine (R). In the R3 repeat, the majority of R2R3-CsMYBs had the basic R3 structure of [-F-(X18)-W-(X18)-W-], in which the first conservative W was substituted by other amino acids, such as phenylalanine (F), isoleucine (I), and leucine (L). The highly conservative sequences in the MYB DNA-binding domain were mainly located at the helix-turn-helix (HTH) motif (between the second and third tryptophan of the two R repeats), which functioned in identifying target sequences.

### 2.2. Phylogenetic Analysis and Putative Function of R2R3-MYB TFs

**Figure 1:**
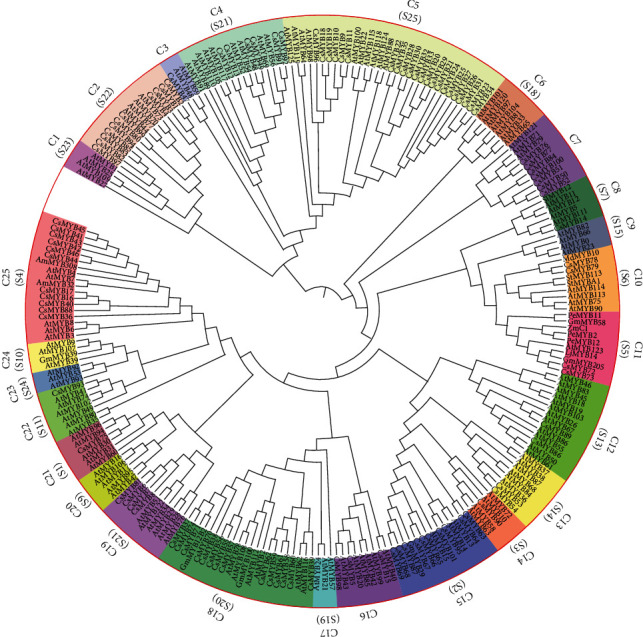
Phylogenetic tree and classification of R2R3-MYB genes among 245 sequences. The neighbor-joining (NJ) tree was constructed using the sequences of 101 R2R3-MYB from *C. speciosa*, 126 from *Arabidopsis*, and 18 from other various species. The English letters with Arabic numbers outside the large red circle indicate the name of each clade. The amino acid sequences of 101 R2R3-CsMYBs were aligned in MEGA 6.0, and the phylogenetic tree was constructed by the NJ method with 1,000 bootstrap replicates. Bootstrap values > 50 are indicated on the nodes. Different clades are marked with different background colors.

Eighteen R2R3-MYB proteins, which have been previously identified to regulate flavonoid biosynthesis as activators/inhibitors (ESM_3) in several plants, and the complete R2R3-MYB subfamily members in *Arabidopsis* (ESM_4) were collected. A neighbor-joining (NJ) unrooted phylogenetic tree was constructed using 101 R2R3-CsMYBs, 126 R2R3-AtMYBs, and 18 function-defined R2R3-MYBs in various plant species (Figure 1). The results showed that 25 clades were subdivided among the 101 CsMYBs based on the topology of the tree (ESM_5). These clades were designated C1 to C25 and separated with different colors, including 7 specific clades (30 proteins) of *A. thaliana* and 18 common clades (213 proteins) between *C. speciosa* and *A. thaliana*.

Homologous proteins that cluster in the same clade presumably possess similar functions. According to the function-annotated R2R3-MYBs from *A. thaliana*, the function of 101 R2R3-CsMYB proteins, belonging to 18 common clades, was predicted. These proteins were mainly divided into five functional groups (ESM_5). Group 1, including 24 proteins in two clades (C5 and C13), was mainly participated in embryogenesis (such as seed, endosperm, and cell differentiation) and development of epidermal cells. Group 2, including 37 proteins in five clades (C2, C15, C18, C21, and C22), was involved in response to biotic and abiotic stresses. Group 3, including 20 proteins in five clades (C8, C10, C11, C19, and C25), was responsible for the regulation of secondary metabolite biosynthesis, such as anthocyanins, glucosinolates, and flavonoids. Interestingly, most (75%) function-defined R2R3-MYB proteins in various plant species were classified to group 3. Group 4, including 9 proteins in three clades (C4, C12, and C14), played important roles in regulating the deposition and regulation of lignin, cellulose, and hemicellulose. Group 5 included 11 proteins in three clades (C3, C7, and C16), whose function was unknown yet. Therefore, these results indicated that R2R3-CsMYBs might play diverse roles in the growth and development, response to stress, and secondary metabolism biosynthesis of *C. speciosa*, in which R2R3-MYB proteins classified to group 3 might be involved in isoflavonoid biosynthesis in *C. speciosa*.

### 2.3. Domain and Motif Composition of *C. speciosa* R2R3-MYB Family Proteins

**Figure 2:**
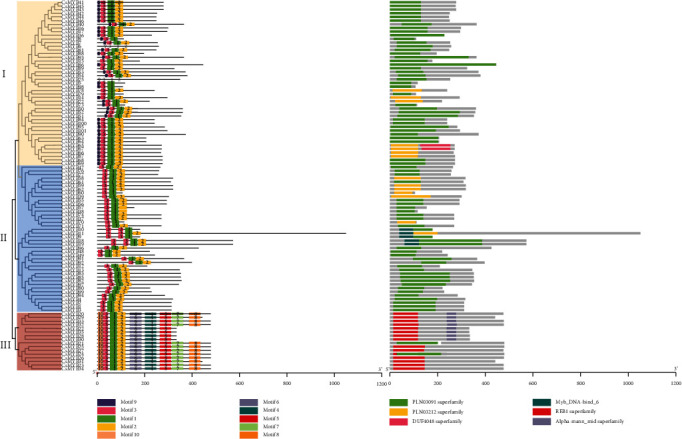
Phylogenetic relationships, motif, and domain compositions of R2R3-MYB genes in *C. speciosa*. The amino acid sequences of 101 R2R3-CsMYBs were aligned in MEGA 6.0, and the phylogenetic tree was constructed by the NJ method with 1,000 bootstrap replicates. Bootstrap values > 50 is indicated on the nodes. Different categories were marked with different background colors. Motifs of R2R3-MYB proteins were analyzed by the MEME web server, and conserved domains were identified by the Pfam 30.0 database, respectively. Each motif/domain is represented by a number on the colored box. The length can be calculated using the scale at the bottom.

**Figure 3:**
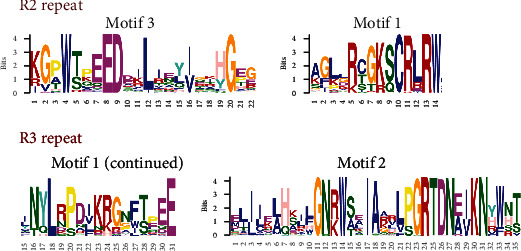
Logo sequences of R2 and R3 repeat of R2R3-MYB subfamily members in *C. speciosa*. The logo sequences of motifs 3, 1, and 2 together constitute the R2 and R3 repeats. The position of each residue and the width of the motif are represented by the Arabic numerals under the colored capital letters, respectively. The overall height of each stack represents the conservation of the sequence at that position. The capital letters indicate greater than 50% conservation of amino acids among the MYB domains. Each color of the letters represents a different type of amino acid residue.

To further investigate the characteristics of the homologous domains among the members of R2R3-CsMYBs (101 proteins), the online MEME tool was applied to analyze the motif distribution regions and the frequencies of the most prevalent amino acids at each position. A maximum of 10 motifs shared by 101 R2R3-CsMYB sequences were selected, and the logo pictures were downloaded from the MEME website (ESM_6). The results suggested that three categories were divided in all R2R3-CsMYB-translated putative protein sequences according to the logo compositions, including I, II, and III. Proteins in category III contained a DNA-binding protein REB1 residue (red), while the others had PLN03091 (green) or PLN03212 (yellow) residues. Among them, motif 3 (pink), motif 1 (green), and motif 2 (yellow) in order appeared in all sequences. Slightly different from the members in categories II and III, and the members in category I harbored motif 9 (purple) in front of motif 3, which belonged to the clades C6–C16 and C19–C25, suggesting that motif 9 might be associated with the DNA-binding site functioned regulation of secondary metabolism. Similarly, 12 out of 101 R2R3-MYB translated putative protein sequences in category III possessed highly conserved motifs 10, 3, 1, 2, 6, 4, 5, 7, and 8 in order, which might be involved in the regulation of embryogenesis (Figure 2). In these R2R3-CsMYBs, motif 3 and the front part of motif 1 were composed of the typical R2 repeat, while the latter part of motif 1 and the entire motif 2 were composed of the typical R3 repeat. Except for the triplet of tryptophan, the R2 and R3 repeats also contained several other conserved residues, such as glutamic acid (E)-aspartic acid (D) residues (ED), leucine (L) residues, and glycine (G) residues (Figure 3).

### 2.4. Expression Analysis of R2R3-CsMYB Genes Based on Transcriptome Data

**Figure 4:**
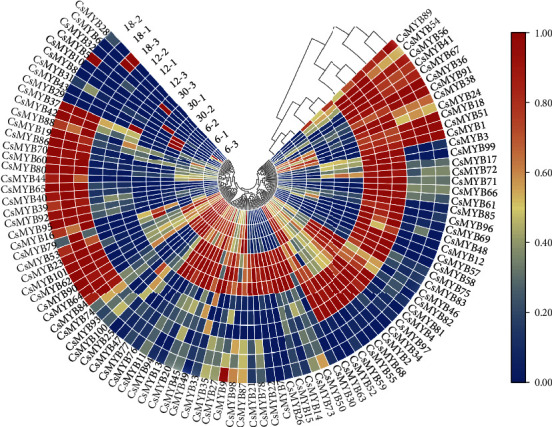
The heat map for R2R3-MYB expression profiles from the transcriptome data at different developmental stages of tuberous roots. Color scale represents log_2_ (FPKM+1) expression values. Changes in the expression level are indicated by a change in color; from blue to red indicates an expression level from low to high.

To assess the potential regulatory function of R2R3-CsMYB genes in isoflavonoid biosynthesis, their expression profiles were analyzed by constructing a heat map using the transcriptome data of *C. speciosa*. The results showed significant differences in the expression profiles of R2R3-CsMYBs among the tuberous roots at four different developmental stages (6, 12, 18, and 30 months after germination (MAG)). Twelve R2R3-CsMYB genes were highly upregulated at both 12 and 18 MAG, such as *CsMYB36*, *CsMYB41*, and *CsMYB56*. Additionally, the expression levels of 23 R2R3-CsMYB genes were specifically upregulated at 18 MAG, whereas 22 genes were highly expressed at 12 MAG. Among the R2R3-CsMYB genes in group 3, fivegenes (*CsMYB16*, *CsMYB40*, *CsMYB42*, *CsMYB44*, and *CsMYB88*) showed high expression at 18 MAG, whereas six genes (*CsMYB7*, *CsMYB14*, *CsMYB45*, *CsMYB73*, *CsMYB78*, and *CsMYB46*) are especially high-expressed at 30 MAG. *CsMYB36* and *CsMYB41* were both highly expressed at 12 and 18 MAG. However, the expression of the other three genes (*CsMYB17*, *CsMYB72*, and *CsMYB81*) was at high levels at 12 MAG (Figure 4). Hence, we speculated that isoflavonoid biosynthesis might be promoted under the complex regulation of these genes during the thickening of *C. speciosa* tuberous roots.

### 2.5. Coexpression Pattern between R2R3-CsMYBs and Isoflavonoid Biosynthetic Genes

**Figure 5:**
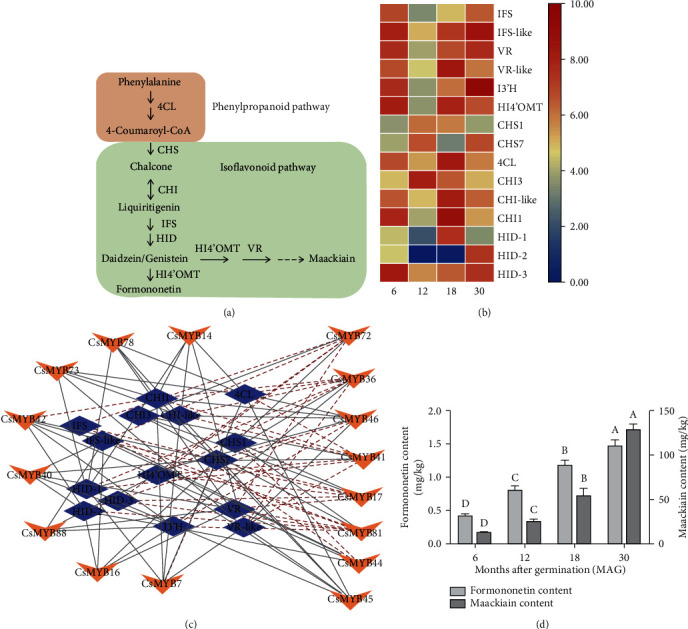
Simplified scheme and a heat map of the expression of key isoflavonoid biosynthetic genes in *C. speciosa*. (a) Isoflavonoid biosynthesis pathway. Enzyme names are indicated for each step. 4CL: 4-coumarate-CoA ligase; CHS: chalcone synthase; CHI: chalcone isomerase; IFS: isoflavone synthase; HID: 2-hydroxyisoflavanone dehydratase; HI4′OMT: isoflavone 4′-O-methyltransferase; VR: vestitone reductase. (b) Heat map of the expression profiles of key isoflavonoid biosynthetic genes. Color scale represents log_2_ (FPKM+1) expression values. Changes in expression level are indicated by a change in color; from blue to red indicates an expression level from low to high. (c) Coexpression networks between key isoflavonoid biosynthetic genes and R2R3-MYBs related to secondary metabolism. The blue purple oval nodes indicate the key isoflavonoid biosynthetic genes, and the orange arrow nodes indicate the R2R3-MYBs. The gray solid lines indicate positive coexpression, and the red dash lines indicate negative coexpression. (d) The content of formononetin and maackiain at four developmental stages (6, 12, 18, and 30 MAG) of tuberous roots. All data shown reflect the mean of three biological replicates (*n* = 3). Means with different letters in each sample represent a significant difference at 0.05 level.

We have previously reported that 15 putative structural genes were involved in isoflavonoid biosynthesis, including *HI4*′*OMT*, 2 *VRs*, 3 *CHIs*, 2 *CHSs*, 3 *HIDs*, 2 *IFSs*, *I3*′*H*, and *4CL* [10]. These genes had different expression patterns among the four different developmental stages (6, 12, 24, and 30 MAG). Two *CHSs* (*CHS1* and *CHS7*) were both highly expressed at 12 MAG. However, more genes showed a low expression level at 12 MAG, such as *HID-1*, *IFSs*, *I3*′*H*, *VRs*, and *HI4*′*OMT* (Figures 5(a) and 5(b)). To determine which TFs may play pivotal roles in isoflavonoid biosynthesis in *C. speciosa*, gene coexpression network analysis was performed between 20 TFs in group 3 and 15 key structural genes involved in the isoflavonoid biosynthesis pathway. The results showed that 16 R2R3-CsMYB genes were coexpressed with these structural genes, in which eight *CsMYBs* had high degrees of coexpression with four isoflavonoid-related genes (*CsIFS*, *CsCHS7*, *CsHID-1*, and *CsCHI3*), indicating their potential contribution to isoflavonoid biosynthesis (Figure 5(c)). *CsMYB36* showed the highest degree of coexpression with isoflavonoid biosynthetic genes, such as *CHI1*, *HID-1*, *HI4*′*OMT*, *VR-like*, and *CHS7*. Further analysis of formononetin and maackiain contents indicated that they were both significantly increased during the development of tuberous roots (Figure 5(d)), indicating that isoflavonoid accumulation might be increased by upregulating both R2R3-CsMYBs and structural genes involved in isoflavonoid biosynthesis. The metabolic flux can be better controlled by coordinated regulation of them.

### 2.6. Validation of Candidate Genes Involved in Isoflavonoid Metabolism

**Figure 6:**
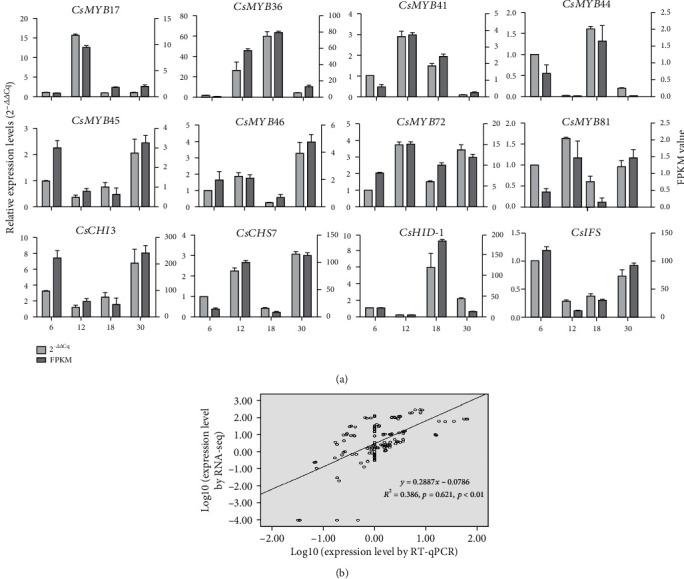
Validation of the transcriptomic data with RT-qPCR. (a) Comparison of the expression levels between RT-qPCR and FPKM values identified by transcriptome. (b) Correlation plot of the RT-qPCR (2^−ΔΔCq^) and FPKM values. The *R*^2^ value represents the correlation between the qPCR and RNA-seq results. Values are the means ± SD of three biological replicates.

The relative expression levels of key genes associated with isoflavonoid accumulation were analyzed to assess the accuracy of the transcriptome sequencing data. Twelve genes including eight TFs and four isoflavonoid biosynthetic genes were selected in this analysis. The results suggested that the RNA sequencing results (FPKM values) were generally consistent with the 2^−ΔΔCq^ values of the selected genes. Significant Pearson correlations between FPKM and 2^−ΔΔCq^ values were also identified in all tested genes (*R*^2^ = 0.386; *p* = 0.621, *p* < 0.01) (Figure 6). Therefore, the RT-qPCR analysis confirmed the validity of the transcriptome data. Meanwhile, the RT-qPCR results showed that there were similar expression profiles between TFs and key structural genes, such as *CsMYB72*, *CsCHS7*, *CsMYB17*, and *CsCHI3*, suggesting that these TFs might activate the promoter activities of key structural genes to regulate isoflavonoid biosynthesis.

## 3. Discussion

The R2R3-MYB subfamily TFs play important roles in hormonal signaling, stress responses, and secondary metabolite accumulation in plants [14, 23]. Flavonoid metabolite biosynthesis is usually activated by R2R3-MYB proteins, including isoflavonoids in soybean (*Glycine max*) [11, 12, 18–20], anthocyanin in *Paeonia suffruticosa* [24], and flavonoids in *Ginkgo biloba* [22]. *C. speciosa* has been used as a folk medicine for hundreds of years in China. Isoflavonoids are the most important active constituents in *C. speciosa* [25]. Isoflavonoids determine the quality of the drugs, and isoflavonoid content varies during the development of tuberous roots [10]. However, the molecular mechanism of isoflavonoid biosynthesis and accumulation that causes variation in isoflavonoid content remains largely unexplored. Therefore, research on R2R3-MYB TFs is helpful for understanding the potential transcriptional genes and their regulation networks of isoflavonoid biosynthesis in *C. speciosa*.

For non-model plants whose genomes have not been sequenced, transcriptome sequencing is a relatively economic and reliable method, providing a dataset for gene screening and transcriptional profiling [26]. Increasing transcription factors have been isolated from plants using a transcriptome dataset, such as barley with 51 R2R3-MYB genes [27], *Ginkgo biloba* with 45 GbMYBs [15], and *Gynostemma pentaphyllum* with 125 GpAP2/ERF [26]. In the present study, 101 R2R3-CsMYBs that harbored conserved domains in *C. speciosa* were identified through transcriptome sequencing (Figure 1). Previous studies revealed that different numbers of R2R3-MYB genes exist in various species. Genome-wide analysis of *Glycine max* and *Medicago truncatula* has identified 244 and 150 R2R3-MYB proteins in legume species, respectively [13, 28]. The number of genes isolated from some plants such as sweet orange (100) [29] and *Salvia miltiorrhiza* (110) [30] was similar, but lower than that of R2R3-MYB proteins in *Arabidopsis* (126) [31]. Although we probably have not identified the whole R2R3-CsMYBs from *C. speciosa* transcriptome data, the identification of these genes can further enrich the resources of *C. speciosa* in GenBank.

The highly conserved tryptophan (W) residues distributed in the third helix are important for DNA-binding activity of MYB proteins, which may indicate functional conservation among different plant species [17, 32]. The members of R2R3-CsMYBs in *C. speciosa* contained the same Trp amino acid residues in the third helix, revealing their highly conserved characteristics (ESM_2). In general, all these proteins possess a highly conserved DNA-binding domain in the N-terminus, and the second half of each R structure is particularly conserved [13, 31]. In the present study, motif 3 (pink), motif 1 (green), and motif 2 (yellow) in order, which consisted of the typical R2 and R3 repeats, appeared in the N-terminus of all R2R3-CsMYB sequences (Figures 2 and 3). Besides them, the members in category I harbored motif 9 distributed in front of motif 3, whereas those in category III possessed a highly conserved motif 10 in front of motif 3. These results indicated that high conservation appeared in the R2R3-CsMYB proteins. The third helical structure of R2R3-CsMYBs was more conserved than the other two helical structures, which was consistent with the findings of previous studies [13, 33]. We assumed that the third helical structure might reflect the functional stability of R2R3-CsMYBs. On the other hand, the species-specific function of R2R3-CsMYBs may result from the variation of the key amino acids in this region. For example, the proline (P) (located on 58) in the linker region was substituted by serine (S) for 16 R2R3-CsMYBs, which was reported to decrease the stability of the protein-DNA complex and even lead to the loss of DNA-binding activity [34, 35]. Additionally, similar to the previous studies [23, 33], the species-specific sequence may distribute in the C-terminus (Figure 2) that distinguishes them from other subfamilies and affects their transcriptional activities. This finding further supported the diversity of the R2R3-CsMYB function.

To better classify the R2R3-CsMYB proteins and predict their functions, a comprehensive analysis of phylogenetic relationships was performed by comparing them with homologous proteins in other species (Figure 1). Based on the presence of distinctive motifs outside of the conserved MYB domains, the majority of the *Arabidopsis* R2R3-type MYBs were classified into 22 subgroups [31]. Due to the function of *Arabidopsis* R2R3-MYB TFs has been well studied and experimentally verified, we predicted the function of R2R3-CsMYBs according to the R2R3-MYB TFs in *A. thaliana*. Seven MYB subgroups have been shown to activate one or more branches of phenylpropanoid metabolism according to the function annotated of R2R3-MYB proteins in *A. thaliana*, including subgroups S3, S4, S5, S6, S7, S12, and S13 [33, 36, 37]. In the present study, 25 clades were subdivided among the 101 R2R3-CsMYBs based on the topology of the tree and the classification of R2R3-MYB TFs, including 7 specific clades (30 proteins) of *A. thaliana* and 18 common clades (213 proteins), indicating a close relationship between *A. thaliana* and *C. speciosa*. The diverse gene structure implied a diverse gene function. Five functional groups were scattered according to the similar functions in this study, including growth and development, response to stress, and secondary metabolism (ESM_5). It is noteworthy that twenty proteins in group 3, including five clades (C8, C10, C11, C19, and C25), harbored motif 9 distributed in front of motif 3 in category I and clustered together with AtMYBs belonging to S4, S5, S6, S7, and S12, indicating that motif 9 might be related to the regulation of flavonoid biosynthesis. The phylogenetic relationships of 18 function-reported R2R3-MYB TFs in various plant species also supported our results, suggesting that the R2R3-CsMYB proteins distributed in group 3 might play important roles in isoflavonoid biosynthesis in *C. speciosa*. The transcriptional function of R2R3-CsMYB proteins in group 3 warrants more detailed investigation.

Transcription factors are often coexpressed with their downstream structural genes. A similar expression pattern is usually an effective way to identify transcription factors regulating secondary metabolism. Several MYB genes in *Pueraria candollei* var. Mirifica (*PmMYB18*, *PmMYB23*, *PmMYB75*, and *PmMYB76*) showed similar expression patterns with the key isoflavonoid biosynthetic genes in tuberous cortices [9]. In potato, *StAN1*, a R2R3-MYB activator, previously named *StAN2* in some studies, was highly expressed in anthocyanin-pigmented tubers and displayed a positive correlation with the transcript levels of structural genes, as well as with anthocyanin content [38]. Our previous studies indicated that *IFSs*, *CHSs*, *HIDs*, *CHIs*, *HI4*′*OMT*, and *VRs* were key isoflavonoid biosynthetic genes [10]. As expected, the expression patterns showed that most R2R3-CsMYBs in group 3 exhibited similar expression patterns with key isoflavonoid biosynthetic genes. Further coexpression analyses identified that eight potential TFs (*CsMYB17*, *CsMYB36*, *CsMYB41*, *CsMYB44*, *CsMYB45*, *CsMYB46*, *CsMYB72*, and *CsMYB81*) were highly coexpressed with four key isoflavonoid biosynthetic genes (*CsIFS*, *CsCHS7*, *CsHID-1*, and *CsCHI3*), consequently promoted formononetin and maackiain accumulation (Figures 4 and 5). The similar expression profile between TFs and key structural genes, such as *CsMYB72*, *CsCHS7*, *CsMYB17*, and *CsCHI3* (Figure 6), suggested that these TFs might act as activators in the promotion of isoflavonoid accumulation in *C. speciosa* roots.

In general, one TF may simultaneously regulate the expression of multiple structural genes and one gene can be simultaneously regulated by multiple TFs [39, 40]. In this study, *CsMYB36* showed the highest degree of coexpression with isoflavonoid biosynthetic genes, such as *CHI1*, *HID-1*, *HI4*′*OMT*, *VR-like*, and *CHS7*. It is interesting to further study how *CsMYB36* interacts with key isoflavonoid biosynthetic genes in *C. speciosa*. Additionally, the flavonoid biosynthetic pathway is transcriptionally regulated by a MBW complex. The activation or repression role of the MBW complex was primarily determined by the MYB transcription factors through binding to the promoters of structural genes, together with the common bHLH and WD40 factors. No information is currently available on how the MBW complex performs its function and what mechanism occurs in *C. speciosa*. To increase the level of isoflavonoid biosynthesis in *C. speciosa*, the MBW complex function should be taken into account in the future.

## 4. Conclusion

The present study was the first transcriptome-wide detailed analysis of R2R3-MYB subfamily genes in *C. speciosa*. A total of 101 R2R3-CsMYBs were identified, which were classified into 25 clades based on their phylogenetic relationships. The deduced R2R3-CsMYB proteins contain highly conserved R2R3 repeat domain at the N-terminal region, that is the signature motif of R2R3-type MYB TFs. The conserved motifs and domain distribution, expression patterns, and coexpression networks help in identifying the potential R2R3-CsMYB TFs in the regulation of isoflavonoid biosynthesis. Eight potential TFs (*CsMYB17*, *CsMYB36*, *CsMYB41*, *CsMYB44*, *CsMYB45*, *CsMYB46*, *CsMYB72*, and *CsMYB81*) had high degrees of coexpression with four key isoflavonoid biosynthetic genes (*CsIFS*, *CsCHS7*, *CsHID-1*, and *CsCHI3*), in which *CsMYB36* as a potential regulator for further investigation showed the highest degree. Further analysis of formononetin and maackiain contents was significantly increased during the development of tuberous roots, indicating that the metabolic flux might be controlled by both related R2R3-CsMYBs and structural genes involved in isoflavonoid biosynthesis. These findings not only potentially accelerate the process of genetic improvement of new cultivars with high isoflavonoid content but also provide novel insights into the molecular regulatory mechanisms of R2R3-CsMYB underlying isoflavonoid biosynthesis in this Chinese medicinal herb with enlarged roots.

## 5. Materials and Methods

### 5.1. Plant Materials

Seedlings of *C. speciosa* were incubated in wet perlite under dark conditions at 26 ± 2°C until germination. Two days after germination, seedlings were cultured in a greenhouse equipped with a system for monitoring temperature (25 ± 4°C), relative humidity (60 ± 5%), and natural photoperiod in the Guangxi Botanical Garden of Medicinal Plants (Nanning, China), with the same cultivation technique measure. Tuberous roots at four developmental stages (6, 12, 18, and 30 MAG) of *C. speciosa* were collected. Three different plants were used as biological replicates. Samples were immediately frozen in liquid nitrogen after collection and stored at -80°C until further use.

### 5.2. Determination of Formononetin and Maackiain Contents

The content of formononetin and maackiain were detected according to the method described by Chen et al. [2]. In brief, diamonsil C_18_ (2) column was used as a chromatographic column. The mobile phase was acetonitrile : 0.1%acetic acid solution = 38 : 62 at a flow rate of 1.0 mL min^−1^. The detection wavelengths were set at 248 and 310 nm. The column temperature was maintained at 35°C with an injection volume of 20 *μ*L. The calibration curve of formononetin was in good linearity over the range of 0.0025−0.0610 *μ*g (*r* = 0.9998). The average recovery was 100.0% with RSD of 2.00% (*n* = 6). The calibration curve of maackiain was in good linearity over the range of 0.0387−1.0152 *μ*g (*r* = 0.9999). The average recovery was 100.6% with RSD of 0.70% (*n* = 6).

### 5.3. RNA Library Construction and Transcriptome Sequencing

Total RNA from roots was extracted according to the TRIzol method (Catalog No. 15596-018; Invitrogen Life Technologies, Carlsbad, California, USA) as described in the manufacturer's instructions. For each sample, an equal amount (20 *μ*g) of total RNA (RIN > 8.0) was applied to construct a cDNA library with the method described by Yao et al. [10]. Finally, twelve libraries (three replicates per sample) were sequenced in pair-end (2 × 100 bp) format BGISEQ-500 sequencing platform, reads were processed and de novo assembled, and the results were analyzed by the BGI Tech Solutions Co., Ltd. (BGI Tech) (BGI, Shenzhen, China).

### 5.4. Analysis of the Transcriptome Data

Adaptor sequences (reads with ambiguous base “N”) and low-quality reads (reads having more than 20% of bases with quality ≤ 15) were filtered. After filtering the raw data, we obtained 153,153 unigenes with a total length of 217.31 Mb and N50 value of 2,167 bp (ESM_7). The filtered clean reads can be found in the NCBI SRA repository, https://www.ncbi.nlm.nih.gov/sra/, with the accession No. SRP223620. The *de novo* assembly, annotation, and enrichment analysis of unigenes were performed according to the method of Yao [10]. The differential expression of unigenes was compared by using the DEGseq R package (1.18.0), with a threshold of FPKM ≥ 10 in each pairwise comparison, ∣log_2_FC (fold change)  | ≥1 and *p* value < 0.05. In addition, multiple testing corrections were performed by controlling the false discovery rate (FDR) to less than 0.05. Finally, the heat map of expression cluster of isoflavonoid-related genes was generated and visualized using TBtools software [41].

### 5.5. Identification of the R2R3-MYB Genes in *C. speciosa*

The Hidden Markov Model (HMM) profile for the MYB binding domain (PF00249) obtained from the Pfam 30.0 database (http://pfam.xfam.org/) [42] was used as a query to search in the *C. speciosa* transcriptome database using HMMER (http://hmmer.org/) [43] with an initial threshold value of *E* ≤ 10^−5^ for selecting ID numbers of all sequences containing MYB domains. A total of 142 contigs were found. All these contigs were downloaded from the transcriptome database and inspected to ensure they contained the conserved domains by SMART version 7 [44] (http://smart.embl-heidelberg.de/) and NCBI's conserved domain database (CDD) [41]. After removing short sequences (<100 bp) and fliting redundant sequences which shared >95% matches [26], the sequences with R2R3-MYB conserved domains were considered to be the R2R3-MYB superfamily members of *C. speciosa*. Then, we got the full-length CDS of 101 R2R3-CsMYB sequences. The physicochemical parameters of these proteins were then further analyzed. The predicted MWs and pIs of R2R3-CsMYB proteins were predicted with ProtParam (http://web.expasy.org/protparam/) [33]. The subcellular location of R2R3-CsMYB proteins was predicted with the PSORT online program (http://www.genscript.com/psort.html). In addition, the sequences of the R2 and R3 MYB repeats in all R2R3-MYB proteins were aligned with the *Clustal X* 2.0 program to visualize their conserved DNA-binding regions. The heat map of the expression cluster of R2R3-MYB genes was visualized using TBtools software [41].

### 5.6. Phylogenetic Tree and Conserved Motif Analysis of MYB Proteins

The multiple sequence alignment (MSA) of 245 R2R3-MYB proteins was performed including 101 R2R3-MYB proteins of *C. speciosa*, 126 from *A. thaliana*, and 18 from other plants. The amino acid sequences of *A. thaliana* were downloaded from the TAIR (http://www.arabidopsis.org/) database [31] (ESM_4). Eighteen proteins were retrieved from NCBI (https://www.ncbi.nlm.nih.gov/guide/protein/), which have been reported to regulate isoflavanoid biosynthesis (ESM_3). The 245 proteins were aligned in MEGA 6.0 for the construction of the phylogenetic tree [45]. The neighbor-joining (NJ) phylogenetic tree was constructed based on the aligned results with the following parameters: Poisson correction, pairwise deletion, and bootstrap analysis with 1,000 replicates. The function of R2R3-CsMYB proteins in *C. speciosa* was classified and predicted according to the phylogenetic tree. The conserved motifs of R2R3-CsMYB proteins were analyzed using Multiple Expectation Maximization for Motif Elicitation 5.1.1 (MEME5.1.1: http://meme-suite.org/tools/meme) [46], with the following parameters: minimum and maximum motif widths 6 and 50, respectively, and the maximum number of motifs 10. Finally, Tbtools software [41] was used to visualize the phylogenetic tree, conserved motifs, and domains of R2R3-CsMYB proteins.

### 5.7. Construction of Coexpression Network

The expression profiles of transcripts between key structural genes and all *R2R3-CsMYBs*, which potentially were responsible for the regulation of secondary metabolism, were used to construct the coexpression network. The expression correlation matrix was generated with Cytoscape v3.5.1 to measure the similarity of expression between pairwise genes. Gene pairs with *r* > 0.60 (positive coexpression) or *r* < −0.60 (negative coexpression) were considered significantly coexpressed.

### 5.8. RT-qPCR Analysis

To validate the expression profiles of candidate TFs and isoflavonoid-related genes in *C. speciosa*, RT-qPCR experiment was performed with SYBR® Premix Ex Taq™ (Bio-Rad Laboratories, Hercules, CA, USA). A total of 1 *μ*g of RNA was reverse-transcribed into cDNA using the PrimeScript RT reagent with the gDNA Eraser kit (TaKaRa, Dalian, China). The cDNA was diluted to five-fold before preparing the reaction system. The RT-qPCR analysis was performed by following the MIQE guidelines [47]. The gene-specific primers were designed using Primer 5.0 (ESM_8) and synthesized by BGI-Shenzhen. Glyceraldehyde-3-phosphate dehydrogenase (*GAPDH*) was selected as an internal reference gene. For each primer, a single amplification of expected length by 1.0% agarose gel electrophoresis and a sole peak in the dissociation curve by RT-qPCR analysis were verified. The amplicon size ranged between 80 bp and 280 bp. The amplification efficiencies of the genes ranged from 95 to 115%. PCR reactions with no-template controls were also performed for each primer pair. The reactions were prepared in a total volume of 20 *μ*L containing 10 *μ*L SYBR® Premix Ex Taq™, 2 *μ*L cDNA, 1 *μ*L each 10 *μ*M primer, and 6 *μ*L ddH_2_O. The real-time PCRs were performed employing Light Cycler® 480II Real-Time System (Roche), according to the supplier's manuals. All the PCRs were performed under the following conditions: one cycle of 15 min at 95°C for activation, followed by 40 cycles of 10 sec at 95°C, 30 sec at 60°C, and 30 sec at 72°C. Three technical replicates were conducted for each of the three biological replicates. The relative expression of the candidate gene was calculated by the 2^−ΔΔCq^ formula [48]. Pearson correlation analysis between FPKM and 2^−*ΔΔ*Cq^ was performed using the R package.

### 5.9. Statistical Analyses

The summary statistics presented in the figures were analyzed using IBM SPSS Statistics 19.0 software (Ehningen, Germany) and presented as the means ± SD of three replicates for each sample. The statistical significance was determined by ANOVA. Values in figures marked with different lowercase and capital letters are significantly different at 0.05 and 0.01 probability levels, respectively. The figures were drawn using the software GraphPad Prism 5 (GraphPad software, San Diego, CA, USA).

## Data Availability

The dataset generated for this study can be found in the NCBI SRA repository, https://www.ncbi.nlm.nih.gov/sra/, with the accession No. SRP223620.

## Supplementary Materials

Supplementary 1 ESM_1: physicochemical properties of R2R3-MYB family members in *C. speciosa*.

Supplementary 2 ESM_2: multiple alignment of the amino acid sequences of 101 R2R3-MYB domains in *C. speciosa*.

Supplementary 3 ESM_3: list of 18 R2R3 MYB proteins/genes from other plants and their reported functions.

Supplementary 4 ESM_4: list of 126 R2R3-MYB proteins/genes in *Arabidopsis*.

Supplementary 5 ESM_5: summary of R2R3-MYB superfamily genes and their predicted function in *C. speciosa*.

Supplementary 6 ESM_6: logo sequences of each motif of 10 conserved motifs shared by proteins of R2R3-MYB subfamilies.

Supplementary 7 ESM_7: summary of the BGISEQ-500 RNA-Seq data analysis.

Supplementary 8 ESM_8: sequences of primers designed for RT-qPCR analysis.
