# Supplementary material for: Identification of Isoflavonoid Biosynthesis-Related R2R3-MYB Transcription Factors in Callerya speciosa (Champ. ex Benth.) Schot Using Transcriptome-Based Gene Coexpression Analysis
Source: Int J Genomics. 2021 May 25;2021:9939403. doi: 10.1155/2021/9939403 (PMC8174187; doi:10.1155/2021/9939403)
Supplement: Supplementary 1 — ESM_1: physicochemical properties of R2R3-MYB family members in C. speciosa. [file 9939403.f1.pdf]

ESM 1 Physicochemical properties of *R2R3-MYB* family members in *C. speciosa*

Nomenclature used for this paper	Pl (aa)	MW (kDa)	pI	α-helix (%)	β-turn (%)
CsMYB1	311	33.70	9.09	27.31	3.88
CsMYB2	313	33.71	9.09	27.23	3.96
CsMYB3	312	33.70	9.09	27.33	3.86
CsMYB4	317	33.72	9.11	24.29	2.84
CsMYB5	116	13.49	9.36	47.41	10.34
CsMYB6	257	29.31	9.61	23.35	5.45
CsMYB7	252	28.76	9.02	34.13	6.75
CsMYB8	110	12.88	9.59	31.82	11.82
CsMYB9	180	21.00	9.49	45.56	7.78
CsMYB10	180	21.01	9.49	45.56	7.78
CsMYB11	1050	116.22	5.27	25.52	2.19
CsMYB12	210	24.11	9.20	42.86	5.71
CsMYB13	345	38.72	6.63	33.62	5.22
CsMYB14	292	33.09	9.08	29.45	7.53
CsMYB15	178	20.75	9.42	29.21	7.87
CsMYB16	297	32.97	8.74	24.24	5.05
CsMYB17	294	32.59	8.74	27.21	4.42
CsMYB18	572	62.95	8.71	26.57	3.15
CsMYB19	572	62.95	8.71	26.57	3.15
CsMYB20	476	53.58	6.42	36.55	4.41
CsMYB21	478	53.85	7.62	37.24	4.28
CsMYB22	333	38.55	8.92	45.05	5.11
CsMYB23	477	53.94	8.69	37.11	4.19
CsMYB24	478	54.06	8.41	38.70	3.97
CsMYB25	478	54.06	8.58	38.28	3.97
CsMYB26	477	53.95	8.58	36.48	3.98
CsMYB27	476	53.88	8.55	36.34	4.41
CsMYB28	334	38.62	9.04	46.11	5.39
CsMYB29	442	49.85	6.05	35.75	4.98
CsMYB30	334	38.61	9.04	44.61	5.69
CsMYB31	442	50.02	8.03	37.56	4.75
CsMYB32	477	53.78	6.42	38.99	3.77
CsMYB33	477	53.67	6.42	39.41	3.98
CsMYB34	477	53.94	8.69	37.11	4.19
CsMYB35	333	38.55	8.92	45.05	5.11
CsMYB36	228	26.26	9.16	23.25	8.33
CsMYB37	269	30.47	8.27	38.29	6.32
CsMYB38	116	13.20	7.66	37.07	11.21
CsMYB39	300	34.22	7.70	29.33	4.00
CsMYB40	363	39.57	9.28	21.76	6.34
CsMYB41	277	30.53	8.78	31.77	4.69
CsMYB42	250	27.77	8.78	40.00	6.40
CsMYB43	277	30.45	8.78	36.10	4.33
CsMYB44	248	27.37	8.79	34.68	6.85
CsMYB45	276	30.52	8.78	22.46	6.16
CsMYB46	249	27.48	8.79	31.33	7.23
CsMYB47	264	30.05	5.66	30.68	6.44
CsMYB48	220	25.76	8.30	33.18	4.55
CsMYB49	242	28.58	8.22	33.47	4.96

CsMYB50	360	40.72	6.20	33.33	4.17
CsMYB51	352	39.80	6.03	36.36	3.98
CsMYB52	358	40.48	5.95	31.56	3.07
CsMYB53	370	41.17	7.59	25.95	5.41
CsMYB54	379	42.28	8.95	24.27	2.64
CsMYB55	292	32.87	8.19	35.27	5.82
CsMYB56	292	32.88	8.49	35.96	7.53
CsMYB57	154	18.02	9.77	27.92	12.99
CsMYB58	317	36.27	5.59	30.28	4.42
CsMYB59	318	23.09	5.80	29.87	4.09
CsMYB60	105	12.16	9.03	31.43	10.48
CsMYB61	308	35.05	5.80	31.17	5.52
CsMYB62	318	36.42	5.80	29.87	4.09
CsMYB63	206	22.99	9.34	28.64	4.37
CsMYB64	206	36.96	9.34	31.55	7.28
CsMYB65	271	30.87	5.27	33.95	1.48
CsMYB66	267	30.53	5.57	32.58	4.49
CsMYB67	271	30.90	5.56	26.94	5.54
CsMYB68	272	31.02	5.40	29.78	4.04
CsMYB69	272	31.01	5.40	29.78	4.04
CsMYB70	111	12.51	6.90	27.93	11.71
CsMYB71	269	29.96	5.99	43.49	4.83
CsMYB72	218	47.42	9.53	44.50	6.42
CsMYB73	114	13.25	9.05	33.33	13.16
CsMYB74	269	30.33	5.57	43.87	4.09
CsMYB75	347	37.82	6.47	32.56	1.44
CsMYB76	258	29.97	8.93	50.78	6.59
CsMYB77	255	29.55	6.91	46.27	5.88
CsMYB78	239	27.54	8.93	38.49	8.79
CsMYB79	109	12.88	9.57	50.46	12.84
CsMYB80	220	24.51	9.36	39.09	3.18
CsMYB81	248	28.04	9.36	26.61	5.65
CsMYB82	353	37.94	5.62	26.35	4.53
CsMYB83	351	37.71	5.50	27.64	4.84
CsMYB84	240	27.56	5.75	32.08	5.42
CsMYB85	351	37.71	5.50	27.64	4.84
CsMYB86	445	49.89	7.53	30.79	3.60
CsMYB87	272	76.59	5.36	32.35	5.15
CsMYB88	196	22.34	9.02	35.71	6.63
CsMYB89	324	36.67	6.23	35.49	4.94
CsMYB90	371	41.69	4.77	30.46	5.39
CsMYB91	365	39.59	8.94	25.75	4.11
CsMYB92	397	42.74	6.86	20.40	4.28
CsMYB93	363	40.77	6.72	32.78	4.96
CsMYB94	283	31.24	6.55	31.45	4.24
CsMYB95	282	32.42	6.71	29.43	3.90
CsMYB96	426	48.07	8.57	22.54	2.58
CsMYB97	343	37.18	5.74	25.07	2.62
CsMYB98	107	12.32	9.40	39.25	12.15
CsMYB99	226	24.73	8.42	36.28	5.75
CsMYB100	240	27.59	5.75	27.92	5.00
CsMYB101	293	32.83	4.85	24.91	5.12

extended strand (%)	random coil (%)	Subcellular localization prediction
6.72	62.09	Nucleus
6.74	62.07	Nucleus
6.75	62.06	Nucleus
7.26	65.62	Nucleus
12.07	30.17	Nucleus
12.06	59.14	Nucleus
15.08	44.05	Nucleus
10.91	45.45	Nucleus
1.11	45.56	Nucleus
1.11	45.56	Nucleus
8.48	63.81	Nucleus
7.14	44.29	Nucleus
10.14	51.01	Nucleus
11.30	51.71	Nucleus
6.74	56.18	Nucleus
9.76	60.94	Nucleus
11.22	57.14	Nucleus
3.85	66.43	Nucleus
3.85	66.43	Nucleus
7.98	51.05	Nucleus
8.37	50.21	Nucleus
8.41	41.44	Nucleus
8.18	50.52	Nucleus
8.37	48.95	Nucleus
8.79	48.95	Nucleus
9.43	50.10	Nucleus
8.61	50.63	Nucleus
8.68	39.82	Nucleus
9.05	50.23	Nucleus
8.08	41.62	Nucleus
8.82	48.87	Nucleus
8.81	48.43	Nucleus
8.81	47.80	Nucleus
8.18	50.52	Nucleus
8.41	41.44	Nucleus
17.98	50.44	Nucleus
7.43	47.96	Nucleus
15.52	36.21	Nucleus
6.67	60.00	Nucleus
8.82	63.09	Nucleus
8.30	55.23	Nucleus
4.80	48.80	Nucleus
7.58	51.99	Nucleus
9.27	49.19	Nucleus
10.51	60.87	Nucleus
7.63	50.82	Nucleus
9.85	53.03	Nucleus
7.73	54.55	Nucleus
10.33	51.24	Nucleus

8.89	53.61	Nucleus
5.68	53.98	Nucleus
6.98	58.38	Nucleus
9.19	59.46	Nucleus
7.65	65.44	Nucleus
7.19	51.71	Nucleus
9.25	47.26	Nucleus
20.78	38.31	Nucleus
7.89	57.41	Nucleus
6.92	59.12	Nucleus
16.19	41.90	Nucleus
5.84	57.47	Nucleus
6.92	59.12	Nucleus
2.43	64.56	Nucleus
3.88	57.28	Nucleus
4.80	59.78	Nucleus
0.00	62.92	Nucleus
4.43	63.10	Nucleus
3.68	62.50	Nucleus
3.68	62.50	Nucleus
16.22	44.14	Nucleus
8.92	42.75	Nucleus
1.83	47.25	Nucleus
2.63	50.88	Nucleus
5.20	46.84	Nucleus
4.61	61.38	Nucleus
5.81	36.82	Nucleus
5.10	42.75	Nucleus
8.37	44.35	Nucleus
5.50	31.19	Nucleus
4.09	53.64	Nucleus
9.68	58.06	Nucleus
10.48	58.64	Nucleus
9.97	57.55	Nucleus
6.25	56.25	Nucleus
9.97	57.55	Nucleus
4.49	61.12	Nucleus
2.57	59.93	Nucleus
7.14	50.51	Nucleus
5.86	53.70	Nucleus
6.47	57.68	Nucleus
10.14	60.00	Nucleus
15.11	60.20	Nucleus
16.25	46.01	Nucleus
11.66	52.65	Nucleus
3.90	62.77	Nucleus
7.51	67.37	Nucleus
9.04	63.27	Nucleus
15.89	32.71	Nucleus
6.64	51.33	Nucleus
4.17	62.92	Nucleus
4.44	65.53	Nucleus
