# Supplementary figures and images for: Identification of Isoflavonoid Biosynthesis-Related R2R3-MYB Transcription Factors in Callerya speciosa (Champ. ex Benth.) Schot Using Transcriptome-Based Gene Coexpression Analysis
Source: Int J Genomics. 2021 May 25;2021:9939403. doi: 10.1155/2021/9939403 (PMC8174187; doi:10.1155/2021/9939403)

in *C. speciosa*.

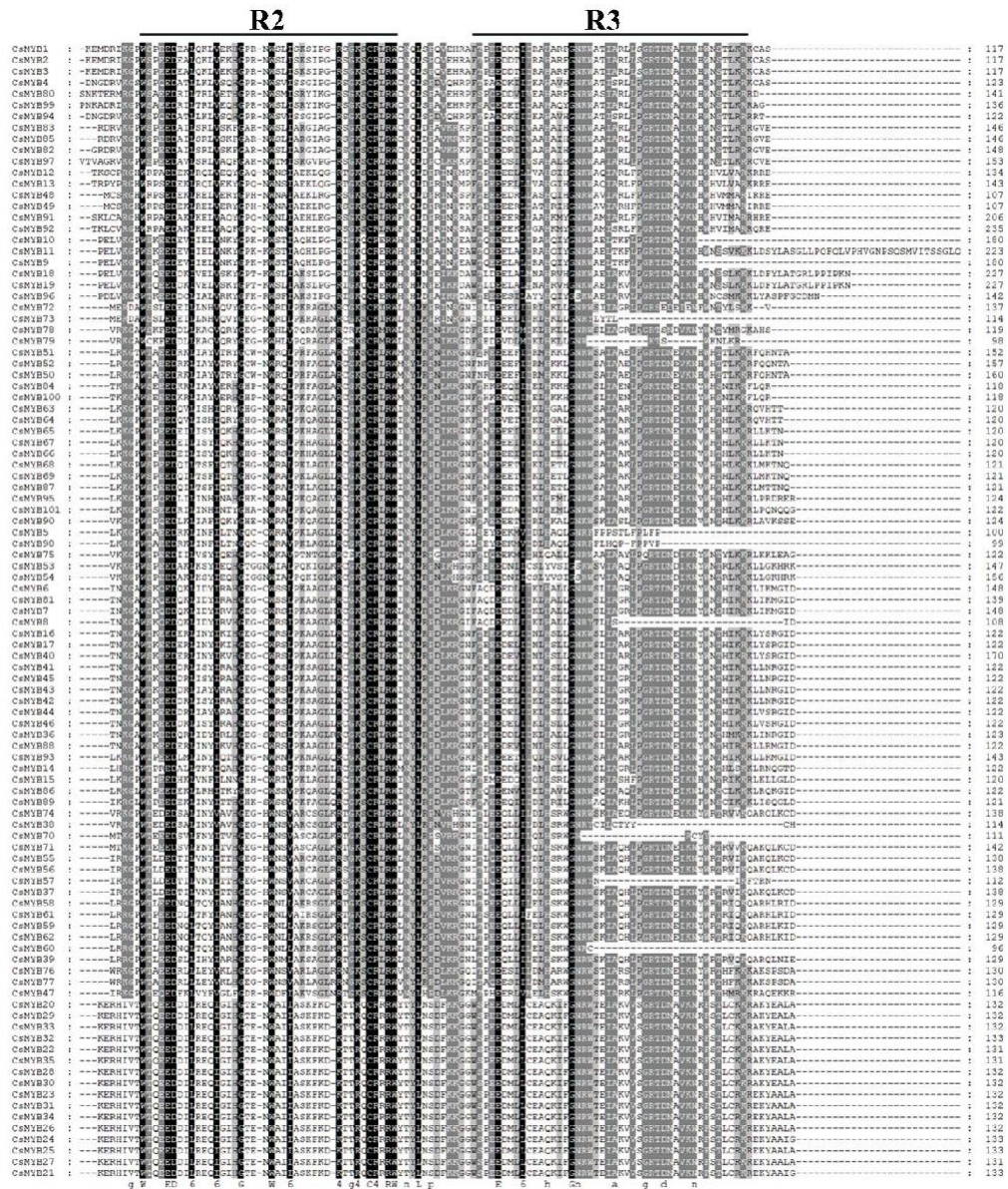

Supplement: Supplementary 2 — ESM_2: multiple alignment of the amino acid sequences of 101 R2R3-MYB domains in C. speciosa. [file 9939403.f2.pdf]
