# Supplementary material for: Identification of Isoflavonoid Biosynthesis-Related R2R3-MYB Transcription Factors in Callerya speciosa (Champ. ex Benth.) Schot Using Transcriptome-Based Gene Coexpression Analysis
Source: Int J Genomics. 2021 May 25;2021:9939403. doi: 10.1155/2021/9939403 (PMC8174187; doi:10.1155/2021/9939403)
Supplement: Supplementary 3 — ESM_3: list of 18 R2R3 MYB proteins/genes from other plants and their reported functions. [file 9939403.f3.pdf]

ESM 3 List of 18 R2R3 MYB proteins/genes from other plants and their reported functions.

**Gene/Protein name**	**ID**	**Species**	**Functions**
AmMYB308	P81393	*Antirrhinum majus*	Activator for phenylpropanoid and lignin biosynthesis
GmMYB58	XP_003525887	*Glycine max*	Activator for isoflavonoid biosynthesis
GmMYB205	XP_003550620	*Glycine max*	Activator for isoflavonoid biosynthesis
GmMYB12B2	AEC13303	*Glycine max*	Activator for flavonoid biosynthesis
GmMYB100	ABH02893	*Glycine max*	Inhibitor for isoflavonoid (soybean) and flavonol (*Arabidopsis*) biosynthesis
GmMYB29	NP_001241360.1	*Glycine max*	Activator for isoflavonoid biosynthesis
GmMYB39	XP_006572975.1	*Glycine max*	Activator for isoflavonoid biosynthesis
GmMYB133	NP_001237536.2	*Glycine max*	Activator for isoflavonoid biosynthesis
GmMYB176	NP_001236048	*Glycine max*	Activator for isoflavonoid biosynthesis
LjMYB14	ARK19306.1	*Lotus corniculatus*	Activator for phenylpropanoid and isoflavonoid biosynthesis
MdMYB10	ACQ45201	*Malus domestica*	Activator for anthocynin biosynthesis
PeMYB2	AIS35919	*Phalaenopsis equestris*	Activator for anthocynin biosynthesis
PeMYB11	AIS35928	*Phalaenopsis equestris*	Activator for anthocynin biosynthesis
PeMYB12	AIS35929	*Phalaenopsis equestris*	Activator for anthocynin biosynthesis
SlMYB12	NP_001234401	*Solanum lycopersicum*	Activator for naringenin chalcone and flavonol biosynthesis
StMYB113	AND01219	*Solanum tuberosum*	Activator for anthocynin biosynthesis
StMYBA1	ALA13582	*Solanum tuberosum*	Activator for anthocynin biosynthesis
ZmC1	1613412E	*Zea mays*	Activator for anthocynin biosynthesis
