# Supplementary material for: Identification of Isoflavonoid Biosynthesis-Related R2R3-MYB Transcription Factors in Callerya speciosa (Champ. ex Benth.) Schot Using Transcriptome-Based Gene Coexpression Analysis
Source: Int J Genomics. 2021 May 25;2021:9939403. doi: 10.1155/2021/9939403 (PMC8174187; doi:10.1155/2021/9939403)
Supplement: Supplementary 4 — ESM_4: list of 126 R2R3-MYB proteins/genes in Arabidopsis. [file 9939403.f4.pdf]

ESM_4 List of 126 R2R3-MYB proteins/genes in *Arabidopsis.*

Locus ID	Gene/Protein name
At3g27920	AtMYB0
At3g09230	AtMYB1
At3g12820	AtMYB10
At2g25230	AtMYB100
At2g32460	AtMYB101
At4g21440	AtMYB102
At1g63910	AtMYB103
At2g26950	AtMYB104
At1g69560	AtMYB105
At3g01140	AtMYB106
At3g02940	AtMYB107
At3g06490	AtMYB108
At3g55730	AtMYB109
At3g62610	AtMYB11
At3g29020	AtMYB110
At5g49330	AtMYB111
At1g48000	AtMYB112
At1g66370	AtMYB113
At1g66380	AtMYB114
At5g40360	AtMYB115
At1g25340	AtMYB116
At1g26780	AtMYB117
At3g27785	AtMYB118
At5g58850	AtMYB119
At2g47460	AtMYB12
At5g55020	AtMYB120
At3g30210	AtMYB121
At1g74080	AtMYB122
At5g35550	AtMYB123
At1g14350	AtMYB124
At3g60460	AtMYB125
At1g06180	AtMYB13
At2g31180	AtMYB14
At3g23250	AtMYB15
At5g15310	AtMYB16
At3g61250	AtMYB17
At4g25560	AtMYB18
At5g52260	AtMYB19
At2g47190	AtMYB2
At1g66230	AtMYB20
At3g27810	AtMYB21
At5g40430	AtMYB22
At5g40330	AtMYB23
At5g40350	AtMYB24
At2g39880	AtMYB25
At3g13890	AtMYB26
At3g53200	AtMYB27
At5g61420	AtMYB28
At5g07690	AtMYB29
At1g22640	AtMYB3
At3g28910	AtMYB30

At1g74650	AtMYB31
At4g34990	AtMYB32
At5g06100	AtMYB33
At5g60890	AtMYB34
At3g28470	AtMYB35
At5g57620	AtMYB36
At5g23000	AtMYB37
At2g36890	AtMYB38
At4g17785	AtMYB39
At4g38620	AtMYB4
At5g14340	AtMYB40
At4g28110	AtMYB41
At4g12350	AtMYB42
At5g16600	AtMYB43
At5g67300	AtMYB44
At3g48920	AtMYB45
At5g12870	AtMYB46
At1g18710	AtMYB47
At3g46130	AtMYB48
At5g54230	AtMYB49
At3g13540	AtMYB5
At1g57560	AtMYB50
At1g18570	AtMYB51
At1g17950	AtMYB52
At5g65230	AtMYB53
At1g73410	AtMYB54
At4g01680	AtMYB55
At5g17800	AtMYB56
At3g01530	AtMYB57
At1g16490	AtMYB58
At5g59780	AtMYB59
At4g09460	AtMYB6
At1g08810	AtMYB60
At1g09540	AtMYB61
At1g68320	AtMYB62
At1g79180	AtMYB63
At5g11050	AtMYB64
At3g11440	AtMYB65
At5g14750	AtMYB66
At3g12720	AtMYB67
At5g65790	AtMYB68
At4g33450	AtMYB69
At2g16720	AtMYB7
At2g23290	AtMYB70
At3g24310	AtMYB71
At1g56160	AtMYB72
At4g37260	AtMYB73
At4g05100	AtMYB74
At1g56650	AtMYB75
At5g07700	AtMYB76
At3g50060	AtMYB77
At5g49620	AtMYB78
At4g13480	AtMYB79

At1g35515	AtMYB8
At5g56110	AtMYB80
At2g26960	AtMYB81
At5g52600	AtMYB82
At3g08500	AtMYB83
At3g49690	AtMYB84
At4g22680	AtMYB85
At5g26660	AtMYB86
At4g37780	AtMYB87
At2g02820	AtMYB88
At5g39700	AtMYB89
At5g16770	AtMYB9
At1g66390	AtMYB90
At2g37630	AtMYB91
At5g10280	AtMYB92
At1g34670	AtMYB93
At3g47600	AtMYB94
At1g74430	AtMYB95
At5g62470	AtMYB96
At4g26930	AtMYB97
At4g18770	AtMYB98
At5g62320	AtMYB99
