# Supplementary material for: Identification of Isoflavonoid Biosynthesis-Related R2R3-MYB Transcription Factors in Callerya speciosa (Champ. ex Benth.) Schot Using Transcriptome-Based Gene Coexpression Analysis
Source: Int J Genomics. 2021 May 25;2021:9939403. doi: 10.1155/2021/9939403 (PMC8174187; doi:10.1155/2021/9939403)
Supplement: Supplementary 5 — ESM_5: summary of R2R3-MYB superfamily genes and their predicted function in C. speciosa. [file 9939403.f5.pdf]

ESM_5 Summary of R2R3-MYB superfamily genes and their predicted function in *C. speciosa* .

Group code	Name of clade or subfamily	Nomenclature used for this paper	Gene ID	Length of CDS	Length of protein sequence	PI (aa)	MW (kDa)	Localization	Corresponding nomenclature for MYB members in *Arabidopsis*	Annotation in Nr database	Predicted function
		CsMYB9	CL11989.Contig1_Al	731	180	10.11	21.01	Nuclear	AtMYB98	PREDICTED: myb-related protein 3R-1-like isoform X1 [*Lupinus angustifolius*]	Embryogenesis (seed, endosperm and cell differentiation)
		CsMYB10	CL11989.Contig2_Al	756	180	10.11	21.01	Nuclear	AtMYB98	PREDICTED: myb-related protein 3R-1-like isoform X1 [Lupinus angustifolius]	Embryogenesis (seed, endosperm and cell differentiation)
		CsMYB11	CL11989.Contig3_Al	3673	1050	5.11	116.24	Nuclear	AtMYB98	PREDICTED: myb-related protein 3R-1-like isoform X2 [*Cicer arietinum*]	Embryogenesis (seed, endosperm and cell differentiation)
		CsMYB18	CL1330.Contig2_All	1258	572	8.51	62.96	Nuclear	AtMYB4	myb-related protein 308-like [*Glycine max*] >KHN31220.1 Myb-related protein 308 [*Glycine soja*] >KRH63931.1 hypothetical protein GLYMA_04G205100 [*Glycine max*]	Embryogenesis (seed, endosperm and cell differentiation)
		CsMYB19	CL13981.Contig1_Al	2314	572	8.51	62.96	Nuclear	AtMYB98	PREDICTED: myb-related protein B [*Cicer arietinum*]	Embryogenesis (seed, endosperm and cell differentiation)
		CsMYB20	CL13981.Contig2_Al	2091	476	6.88	53.59	Nuclear	AtMYB98	PREDICTED: myb-related protein B [*Cicer arietinum*]	Embryogenesis (seed, endosperm and cell differentiation)
		CsMYB21	CL1416.Contig10_Al	2415	478	7.82	53.86	Nuclear	AtMYB124	transcription factor MYB124 isoform X1 [*Cajanus cajan*]	Embryogenesis (seed, endosperm and cell differentiation)
		CsMYB22	CL1416.Contig11_Al	1347	333	9.11	38.56	Nuclear	AtMYB88	transcription factor MYB124 isoform X1 [*Cajanus cajan*]	Embryogenesis (seed, endosperm and cell differentiation)
		CsMYB23	CL1416.Contig12_Al	2892	477	8.69	53.95	Nuclear	AtMYB124	transcription factor MYB124 isoform X1 [*Cajanus cajan*]	Embryogenesis (seed, endosperm and cell differentiation)
		CsMYB24	CL1416.Contig13_Al	2352	478	8.32	54.07	Nuclear	AtMYB124	transcription factor MYB124 isoform X1 [*Cajanus cajan*]	Embryogenesis (seed, endosperm and cell differentiation)
		CsMYB25	CL1416.Contig14_Al	2303	478	8.54	54.07	Nuclear	AtMYB124	transcription factor MYB124 isoform X1 [*Cajanus cajan*]	Embryogenesis (seed, endosperm and cell differentiation)
	C5 (S25)	CsMYB26	CL1416.Contig15_Al	2879	477	8.54	53.96	Nuclear	AtMYB124	transcription factor MYB124 isoform X1 [*Cajanus cajan*]	Embryogenesis (seed, endosperm and cell differentiation)
1		CsMYB27	CL1416.Contig16_Al	2426	476	8.47	53.9	Nuclear	AtMYB124	transcription factor MYB124-like isoform X1 [*Cajanus cajan*] >XP_020214325.1 transcription factor MYB124-like isoform X1 [*Cajanus cajan*] >XP_020214334.1 transcription factor MYB124-like isoform X2 [*Cajanus cajan*]	Embryogenesis (seed, endosperm and cell differentiation)
		CsMYB28	CL1416.Contig1_All	2787	334	9.4	38.63	Nuclear	AtMYB124	transcription factor MYB124 isoform X1 [*Cajanus cajan*]	Embryogenesis (seed, endosperm and cell differentiation)
		CsMYB29	CL1416.Contig2_All	1810	442	6.45	49.86	Nuclear	AtMYB88	PREDICTED: transcriptional activator Myb-like isoform X1 [*Cicer arietinum*]	Embryogenesis (seed, endosperm and cell differentiation)
		CsMYB30	CL1416.Contig3_All	2009	334	9.4	38.61	Nuclear	AtMYB88	PREDICTED: transcriptional activator Myb-like isoform X1 [*Cicer arietinum*]	Embryogenesis (seed, endosperm and cell differentiation)
		CsMYB31	CL1416.Contig4_All	1608	442	8.08	50.03	Nuclear	AtMYB88	PREDICTED: transcriptional activator Myb-like isoform X1 [*Cicer arietinum*]	Embryogenesis (seed, endosperm and cell differentiation)
		CsMYB32	CL1416.Contig5_All	2009	477	6.88	53.8	Nuclear	AtMYB88	transcription factor MYB124 isoform X1 [*Cajanus cajan*]	Embryogenesis (seed, endosperm and cell differentiation)
		CsMYB33	CL1416.Contig6_All	2468	477	6.88	53.68	Nuclear	AtMYB124	transcription factor MYB124 isoform X1 [*Cajanus cajan*]	Embryogenesis (seed, endosperm and cell differentiation)
		CsMYB34	CL1416.Contig7_All	2915	477	8.69	53.95	Nuclear	AtMYB124	PREDICTED: transcriptional activator Myb-like isoform X1 [*Cicer arietinum*]	Embryogenesis (seed, endosperm and cell differentiation)
		CsMYB35	CL1416.Contig8_All	3293	333	9.11	38.56	Nuclear	AtMYB124	transcription factor MYB124 isoform X1 [*Cajanus cajan*]	Embryogenesis (seed, endosperm and cell differentiation)
		CsMYB96	Unigene39024_All	1512	426	8.33	48.07	Nuclear	AtMYB98	PREDICTED: transcription factor MYB76-like isoform X2 [*Cicer arietinum*]	Embryogenesis (seed, endosperm and cell differentiation)
	C13 (S14)	CsMYB53	CL2723.Contig1_All	1255	370	7.76	41.18	Nuclear	AtMYB84	myb transcription factor [*Medicago truncatula*] >KEH39973.1 myb transcription factor [*Medicago truncatula*]	Epidermal cells
		CsMYB54	CL2723.Contig2_All	1410	379	8.9	42.29	Nuclear	AtMYB84	myb transcription factor [*Medicago truncatula*] >KEH39973.1 myb transcription factor [*Medicago truncatula*]	Epidermal cells
		CsMYB1	CL10294.Contig1_Al	1911	311	9.27	33.71	Nuclear	AtMYB73	PREDICTED: transcription factor MYB44-like [*Cicer arietinum*]	Responses to biotic and abiotic stresses
		CsMYB2	CL10294.Contig2_Al	2025	311	9.27	33.71	Nuclear	AtMYB73	PREDICTED: transcription factor MYB44-like [*Cicer arietinum*]	Responses to biotic and abiotic stresses
		CsMYB3	CL10294.Contig3_Al	2167	311	9.27	33.71	Nuclear	AtMYB73	PREDICTED: transcription factor MYB44-like [*Cicer arietinum*]	Responses to biotic and abiotic stresses
		CsMYB4	CL10294.Contig4_Al	1768	317	9.28	33.72	Nuclear	AtMYB73	PREDICTED: transcription factor MYB44-like [*Cicer arietinum*]	Responses to biotic and abiotic stresses
		CsMYB80	Unigene1103_All	1008	220	9.59	24.51	Nuclear	AtMYB73	PREDICTED: transcription factor MYB44-like [*Ziziphus jujuba*]	Responses to biotic and abiotic stresses
	C2(S22)	CsMYB82	Unigene12129_All	1707	353	5.7	37.94	Nuclear	AtMYB1	transcription factor MYB1 [*Cajanus cajan*]	Responses to biotic and abiotic stresses
		CsMYB83	Unigene18033_All	2961	351	5.47	37.71	Nuclear	AtMYB1	transcription factor MYB1 [*Cajanus cajan*]	Responses to biotic and abiotic stresses
		CsMYB85	Unigene18574_All	2518	351	5.47	37.71	Nuclear	AtMYB1	transcription factor MYB1 [*Cajanus cajan*]	Responses to biotic and abiotic stresses
		CsMYB94	Unigene30903_All	1162	283	7.04	31.25	Nuclear	AtMYB73	PREDICTED: transcription factor MYB44-like [*Cicer arietinum*]	Responses to biotic and abiotic stresses
		CsMYB97	Unigene42113_All	1614	343	6.01	37.15	Nuclear	AtMYB109	transcription factor MYB1-like [*Cajanus cajan*] >KYP48727.1 Transcription factor MYB44 [*Cajanus cajan*]	Responses to biotic and abiotic stresses
		CsMYB99	Unigene45628_All	1503	226	8.35	24.73	Nuclear	AtMYB77	PREDICTED: transcriptional activator Myb-like [*Cicer arietinum*]	Responses to biotic and abiotic stresses
		CsMYB63	CL4544.Contig1_All	997	206	9.68	22.99	Nuclear	AtMYB14	PREDICTED: myb-related protein Myb4-like [*Glycine max*] >KHN14820.1 Myb-related protein Myb4 [*Glycine soja*] >KRH34142.1 hypothetical protein GLYMA_10G165800 [*Glycine max*]	Response to cold stress
		CsMYB64	CL4544.Contig2_All	1107	206	9.68	23.1	Nuclear	AtMYB14	PREDICTED: myb-related protein Myb4-like [*Glycine max*] >KHN14820.1 Myb-related protein Myb4 [*Glycine soja*] >KRH34142.1 hypothetical protein GLYMA_10G165800 [*Glycine max*]	Response to cold stress
		CsMYB65	CL6186.Contig1_All	1438	271	5.1	30.87	Nuclear	AtMYB14	PREDICTED: myb-related protein Myb4-like [*Cicer arietinum*]	Response to cold stress
		CsMYB66	CL6186.Contig2_All	1463	267	5.64	30.53	Nuclear	AtMYB15	PREDICTED: myb-related protein Myb4-like [*Cicer arietinum*]	Response to cold stress
	C15 (S2)	CsMYB67	CL6186.Contig3_All	1267	271	5.64	30.9	Nuclear	AtMYB15	PREDICTED: myb-related protein Myb4-like [*Cicer arietinum*]	Response to cold stress
		CsMYB68	CL6186.Contig5_All	1385	272	5.25	31.2	Nuclear	AtMYB15	transcription factor MYB14-like [*Cajanus cajan*] >KYP74659.1 Myb-related protein Myb4 [*Cajanus cajan*]	Response to cold stress
		CsMYB69	CL6186.Contig6_All	1376	272	5.25	31.2	Nuclear	AtMYB15	transcription factor MYB14-like [*Cajanus cajan*] >KYP74659.1 Myb-related protein Myb4 [*Cajanus cajan*]	Response to cold stress
		CsMYB87	Unigene21235_All	1431	272	5.23	30.89	Nuclear	AtMYB15	transcription factor MYB14-like [*Cajanus cajan*] >KYP74659.1 Myb-related protein Myb4 [*Cajanus cajan*]	Response to cold stress
2		CsMYB95	Unigene36413_All	1037	282	7.17	32.43	Nuclear	AtMYB14	MYB [*Ammopiptanthus nanus*]	Response to cold stress
		CsMYB101	Unigene699_All	1257	293	4.63	32.84	Nuclear	AtMYB15	myb transcription factor [*Medicago truncatula*] >KEH23924.1 myb transcription factor [*Medicago truncatula*]	Response to cold stress
		CsMYB55	CL3302.Contig1_All	1551	292	8.15	32.88	Nuclear	AtMYB2	PREDICTED: transcription factor MYB108-like [*Cicer arietinum*]	Stress responses
		CsMYB56	CL3302.Contig2_All	1610	292	8.38	32.89	Nuclear	AtMYB2	PREDICTED: transcription factor MYB108-like [*Cicer arietinum*]	Stress responses

	CsMYB57	CL3302.Contig9_All	1979	154	10.1	18.03	Nuclear	AtMYB21	PREDICTED: transcription factor MYB108-like [*Cicer arietinum*]	Stress responses
	CsMYB58	CL4313.Contig1_All	1831	317	5.67	36.28	Nuclear	AtMYB116	Transcription factor MYB21 [*Glycine soja*]	Stress responses
	CsMYB59	CL4313.Contig2_All	2062	318	6.1	36.43	Nuclear	AtMYB116	MYB transcription factor MYB84 [*Glycine max*] >ABH02839.1 MYB transcription factor MYB84 [*Glycine max*] >KHN21571.1 Transcription factor MYB21 [*Glycine soja*] >KRH60346.1 hypothetical protein GLYMA_05G234600 [*Glycine max*] >APA28811.1 R2R3-type MYB transcription factor MYB84 [*Glycine max*]	Stress responses
C18 (S20)	CsMYB60	CL4313.Contig4_All	2404	105	9.21	12.17	Nuclear	AtMYB116	MYB transcription factor MYB84 [*Glycine max*] >ABH02839.1 MYB transcription factor MYB84 [*Glycine max*] >KHN21571.1 Transcription factor MYB21 [*Glycine soja*] >KRH60346.1 hypothetical protein GLYMA_05G234600 [*Glycine max*] >APA28811.1 R2R3-type MYB transcription factor MYB84 [*Glycine max*]	Stress responses
	CsMYB61	CL4313.Contig5_All	1957	308	6.1	35.06	Nuclear	AtMYB62	PREDICTED: transcription repressor MYB6-like [*Glycine max*] >KRH48497.1 hypothetical protein GLYMA_07G092800 [*Glycine max*]	Stress responses
	CsMYB62	CL4313.Contig6_All	2254	318	6.1	36.43	Nuclear	AtMYB116	MYB transcription factor MYB84 [*Glycine max*] >ABH02839.1 MYB transcription factor MYB84 [*Glycine max*] >KHN21571.1 Transcription factor MYB21 [*Glycine soja*] >KRH60346.1 hypothetical protein GLYMA_05G234600 [*Glycine max*] >APA28811.1 R2R3-type MYB transcription factor MYB84 [*Glycine max*]	Stress responses
	CsMYB39	CL14201.Contig5_Al	1276	300	7.93	34.23	Nuclear	AtMYB2	PREDICTED: transcription factor MYB108-like [*Cicer arietinum*]	Stress responses
	CsMYB37	CL14201.Contig1_Al	1185	269	8.22	30.48	Nuclear	AtMYB2	PREDICTED: transcription factor MYB108-like [*Cicer arietinum*]	Stress responses
	CsMYB38	CL14201.Contig2_Al	1327	116	7.84	13.2	Nuclear	AtMYB112	PREDICTED: transcription factor MYB108-like [*Cicer arietinum*]	Stress responses
	CsMYB70	CL6970.Contig3_All	1518	111	7.51	12.51	Nuclear	AtMYB57	PREDICTED: transcription factor MYB108-like [*Vigna radiata* var. radiata]	Stress responses
	CsMYB71	CL6970.Contig4_All	1478	269	6.41	29.96	Nuclear	AtMYB2	transcription factor MYB108-like [*Cajanus cajan*]	Stress responses
	CsMYB74	CL1416.Contig9_All	1379	269	5.65	30.34	Nuclear	AtMYB88	transcription factor MYB124 isoform X2 [*Cajanus cajan*]	Stress responses
C21 (S1)	CsMYB75	CL9164.Contig2_All	1361	347	6.9	37.83	Nuclear	AtMYB30	myb-related protein 306-like isoform X1 [*Cajanus cajan*]	Environmental stress
C22(S11)	CsMYB93	Unigene29051_All	1241	363	7.1	40.78	Nuclear	AtMYB102	PREDICTED: transcription factor MYB39-like [*Vigna radiata* var. radiata]	Respond to biotic stress
C8 (S7)	CsMYB14	CL12323.Contig1_Al	1141	292	9.46	33.1	Nuclear	AtMYB4	PREDICTED: transcription repressor MYB6-like [*Vigna angularis*] >KOM52988.1 hypothetical protein LR48_Vigan09g164700 [*Vigna angularis*] >BAT87828.1 hypothetical protein VIGAN_05124000 [*Vigna angularis* var. angularis]	Flavonol biosynthesis
C10(S6)	CsMYB78	CL9580.Contig1_All	1268	239	9.16	27.54	Nuclear	AtMYB113	transcription factor MYB114-like [*Cajanus cajan*] >KYP64685.1 Transcription factor MYB113 family [*Cajanus cajan*]	Anthocyanin biosynthesis
	CsMYB79	CL9580.Contig2_All	460	109	10.09	12.88	Nuclear	AtMYB113	PREDICTED: transcription factor MYB114-like [*Cicer arietinum*]	Anthocyanin biosynthesis
C11 (S5)	CsMYB72	CL7935.Contig1_All	1628	218	10.13	24.73	Nuclear	AtMYB5	transcription factor MYBZ1 [*Glycine max*] >KRH12515.1 hypothetical protein GLYMA_15G176000 [*Glycine max*]	Biosynthesis of proanthocyanidins (PAs)
	CsMYB73	CL7935.Contig3_All	448	114	9.2	13.25	Nuclear	AtMYB5	PREDICTED: myb-related protein Zm38-like [*Ziziphus jujuba*]	Biosynthesis of proanthocyanidins (PAs)
	CsMYB6	CL11586.Contig1_Al	803	257	10.06	29.32	Nuclear	AtMYB3	Transcription repressor MYB4-like protein [*Glycine soja*]	Glucosinolate biosynthesis
C19 (S12)	CsMYB7	CL11586.Contig2_Al	1135	252	8.96	28.77	Nuclear	AtMYB3	PREDICTED: transcription repressor MYB4-like [*Vigna angularis*] >KOM47020.1 hypothetical protein LR48_Vigan07g072400 [*Vigna angularis*] >BAT81235.1 hypothetical protein VIGAN_03091100 [*Vigna angularis* var. angularis]	Glucosinolate biosynthesis
	CsMYB8	CL11586.Contig4_Al	353	110	9.98	12.88	Nuclear	AtMYB3	Transcription repressor MYB4-like protein [*Glycine soja*]	Glucosinolate biosynthesis
	CsMYB81	Unigene1198_All	851	248	9.7	28.04	Nuclear	AtMYB6	Transcription repressor MYB4-like protein [*Glycine soja*]	Glucosinolate biosynthesis
	CsMYB41	CL14875.Contig2_Al	1327	277	8.57	30.53	Nuclear	AtMYB7	myb-related protein 308-like [*Glycine max*] >KHN31220.1 Myb-related protein 308 [*Glycine soja*] >KRH63931.1 hypothetical protein GLYMA_04G205100 [*Glycine max*]	Anthocyanin biosynthesis
	CsMYB42	CL1594.Contig1_All	534	250	8.56	27.77	Nuclear	AtMYB4	Myb-related protein 315 [*Glycine soja*]	Anthocyanin biosynthesis
	CsMYB43	CL1594.Contig2_All	1314	277	8.57	30.46	Nuclear	AtMYB4	R2R3 MYB transcription factor 4 [*Caragana korshinskii*]	Anthocyanin biosynthesis
	CsMYB44	CL1594.Contig3_All	1234	248	8.56	27.37	Nuclear	AtMYB4	R2R3 MYB transcription factor 4 [*Caragana korshinskii*]	Anthocyanin biosynthesis
	CsMYB45	CL1594.Contig4_All	1237	276	8.57	30.52	Nuclear	AtMYB4	R2R3 MYB transcription factor 4 [*Caragana korshinskii*]	Anthocyanin biosynthesis
C25 (S4)	CsMYB46	CL1594.Contig5_All	1335	249	8.56	27.49	Nuclear	AtMYB4	PREDICTED: myb-related protein 308-like [*Vigna angularis*] >KOM42356.1 hypothetical protein LR48_Vigan04g255400 [*Vigna angularis*]	Anthocyanin biosynthesis
	CsMYB16	CL12508.Contig1_Al	1428	297	8.54	32.98	Nuclear	AtMYB40	R2R3 MYB transcription factor 4 [*Caragana korshinskii*]	Anthocyanin biosynthesis
	CsMYB36	CL9012.Contig1_All	1168	228	9.13	26.26	Nuclear	AtMYB4	MYB-related transcription factor [*Salvia miltiorrhiza*] >AGN52162.1 MYB-related transcription factor [*Salvia miltiorrhiza*]	Anthocyanin biosynthesis
	CsMYB88	Unigene24129_All	732	196	8.89	22.34	Nuclear	AtMYB102	MYB/HD-like transcription factor, partial [*Glycine max*]	Anthocyanin biosynthesis
	CsMYB17	CL1330.Contig1_All	1392	294	8.54	32.59	Nuclear	AtMYB4	myb-related protein 308-like [*Glycine max*] >KHN31220.1 Myb-related protein 308 [*Glycine soja*] >KRH63931.1 hypothetical protein GLYMA_04G205100 [*Glycine max*]	Anthocyanin biosynthesis
	CsMYB40	CL14532.Contig1_Al	1377	363	9.47	39.57	Nuclear	AtMYB62	myb-related protein 340 [*Cajanus cajan*] >KYP75157.1 Transcription factor MYB21 [*Cajanus cajan*]	Anthocyanin biosynthesis
	CsMYB12	CL12228.Contig18_A	634	210	9.47	24.12	Nuclear	AtMYB52	PREDICTED: transcription factor MYB36-like [*Lupinus angustifolius*] >OIW14468.1 hypothetical protein TanjilG_19884 [*Lupinus angustifolius*]	Lignin, xylan and cellulose biosynthesis
	CsMYB13	CL12228.Contig19_A	1246	345	7.12	38.73	Nuclear	AtMYB52	myb transcription factor [*Medicago truncatula*] >KEH35402.1 myb transcription factor [*Medicago truncatula*]	Lignin, xylan and cellulose biosynthesis
C4 (S21)	CsMYB48	CL1854.Contig13_Al	1136	220	8.24	25.76	Nuclear	AtMYB48	Transcription factor MYB59 [*Cajanus cajan*]	Lignin, xylan and cellulose biosynthesis
	CsMYB49	CL2222.Contig1_All	1185	242	8.19	28.59	Nuclear	AtMYB52	transcription factor MYB52-like isoform X1 [*Cajanus cajan*]	Lignin, xylan and cellulose biosynthesis
	CsMYB91	Unigene27912_All	2170	365	8.9	39.6	Nuclear	AtMYB56	transcription factor MYB56-like [*Cajanus cajan*]	Lignin, xylan and cellulose biosynthesis
	CsMYB92	Unigene27913_All	1947	397	7.31	42.74	Nuclear	AtMYB56	Transcription factor MYB44 [*Cajanus cajan*]	Lignin, xylan and cellulose biosynthesis
C12 (S13)	CsMYB86	Unigene20891_All	2026	445	7.63	49.9	Nuclear	AtMYB55	PREDICTED: transcription factor MYB29 [*Vigna angularis*] >XP_017412488.1 PREDICTED: transcription factor MYB29 [*Vigna angularis*] >KOM35384.1 hypothetical protein LR48_Vigan02g153400 [*Vigna angularis*] >BAT95209.1 hypothetical protein VIGAN_08188800 [*Vigna angularis* var. angularis]	Lignin biosynthesis
	CsMYB89	Unigene24528_All	1084	324	6.69	36.67	Nuclear	AtMYB67	transcription factor MYB86-like [*Cajanus cajan*]	Lignin biosynthesis

C14 (S3)	CsMYB90	Unigene24590_All	1507	371	4.49	41.69	Nuclear	AtMYB63	Myb-related protein Zm1 [*Glycine soja*]	Lignin biosynthesis
C3	CsMYB47	CL10294.Contig1_Al	1911	264	5.87	30.05	Nuclear	AtMYB73	PREDICTED: transcription factor MYB44-like [*Cicer arietinum*]	Unknown
	CsMYB76	CL9518.Contig1_All	931	258	9.08	29.97	Nuclear	AtMYB79	Transcription factor MYB48 [*Glycine soja*]	Unknown
	CsMYB77	CL9518.Contig2_All	1314	255	7.45	29.55	Nuclear	AtMYB79	R2R3-MYB factor [*Lablab purpureus*]	Unknown
	CsMYB84	Unigene18398_All	1025	240	6.05	27.56	Nuclear	AtMYB9	PREDICTED: myb-related protein Myb4-like [*Glycine max*] >KRH56056.1 hypothetical protein GLYMA_06G299900 [*Glycine max*]	Unknown
C7	CsMYB100	Unigene6882_All	2952	240	6.05	27.59	Nuclear	AtMYB9	PREDICTED: myb-related protein Myb4-like [*Glycine max*] >KRH56056.1 hypothetical protein GLYMA_06G299900 [*Glycine max*]	Unknown
	CsMYB50	CL2222.Contig2_All	1251	360	6.65	40.72	Nuclear	AtMYB52	myb transcription factor [*Medicago truncatula*] >AES76277.2 myb transcription factor [*Medicago truncatula*]	Unknown
	CsMYB51	CL2322.Contig2_All	1296	352	6.42	39.81	Nuclear	AtMYB14	transcription factor MYB14-like isoform X1 [*Cajanus cajan*] >KYP50300.1 Myb-related protein Myb4 [*Cajanus cajan*]	Unknown
	CsMYB52	CL2322.Contig3_All	1376	358	6.35	40.49	Nuclear	AtMYB14	transcription factor MYB14-like isoform X1 [*Cajanus cajan*] >KYP50300.1 Myb-related protein Myb4 [*Cajanus cajan*]	Unknown
	CsMYB5	CL11081.Contig1_Al	679	116	9.4	13.49	Nuclear	AtMYB20	myb transcription factor [*Medicago truncatula*] >AES99236.1 myb transcription factor [*Medicago truncatula*]	Unknown
C16	CsMYB98	Unigene45467_All	623	107	9.49	12.33	Nuclear	AtMYB20	myb transcription factor [*Medicago truncatula*] >AES99236.1 myb transcription factor [*Medicago truncatula*]	Unknown
	CsMYB15	CL12323.Contig2_Al	1195	178	9.95	20.75	Nuclear	AtMYB4	PREDICTED: transcription repressor MYB6-like [*Vigna angularis*] >KOM52988.1 hypothetical protein LR48_Vigan09g164700 [*Vigna angularis*] >BAT87828.1 hypothetical protein VIGAN_05124000 [*Vigna angularis* var. angularis]	Unknown
