# Supplementary material for: Identification of Isoflavonoid Biosynthesis-Related R2R3-MYB Transcription Factors in Callerya speciosa (Champ. ex Benth.) Schot Using Transcriptome-Based Gene Coexpression Analysis
Source: Int J Genomics. 2021 May 25;2021:9939403. doi: 10.1155/2021/9939403 (PMC8174187; doi:10.1155/2021/9939403)

ESM_6 Logo sequences of each motif of 10 conserved motifs shared by proteins of R2R3-MYB subfamilies.

Motif 1

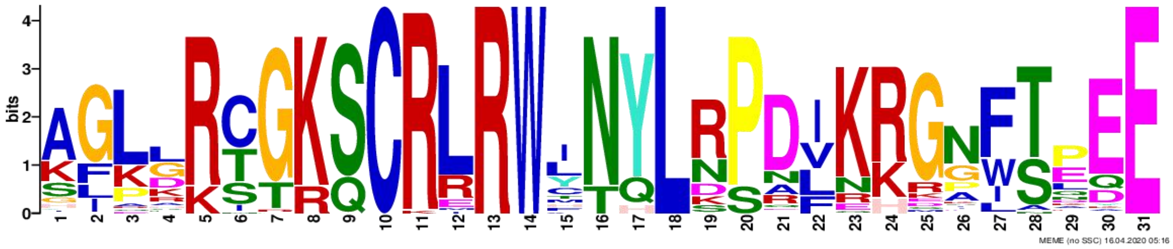

Motif 2

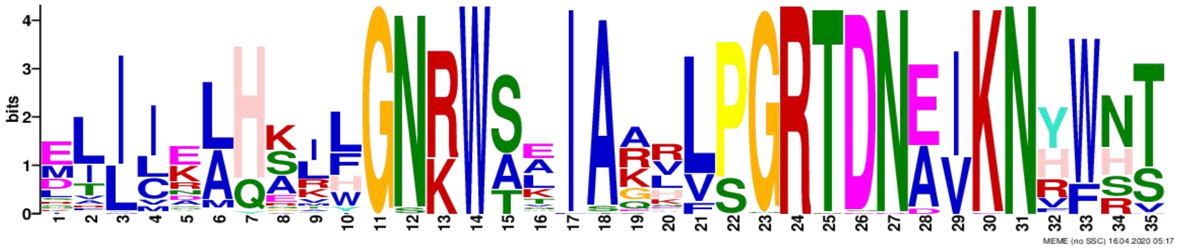

Motif 3

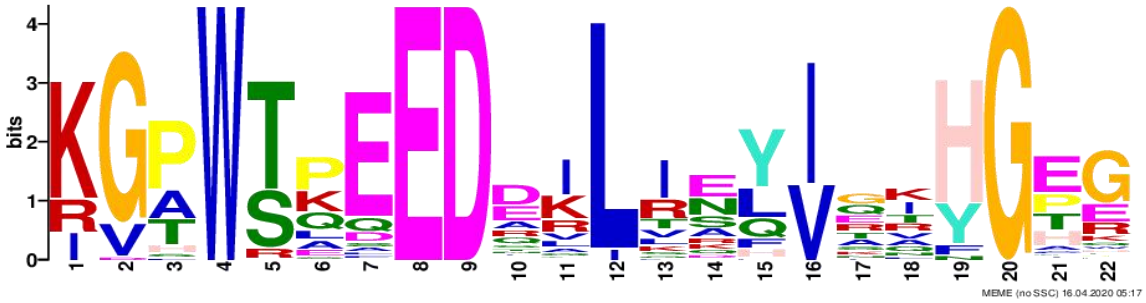

Motif 4

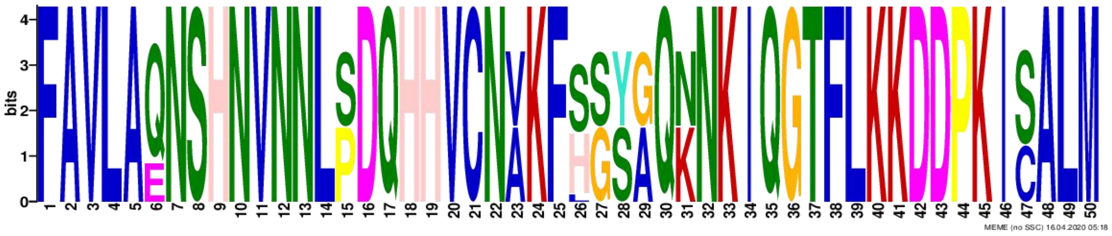

Motif 5

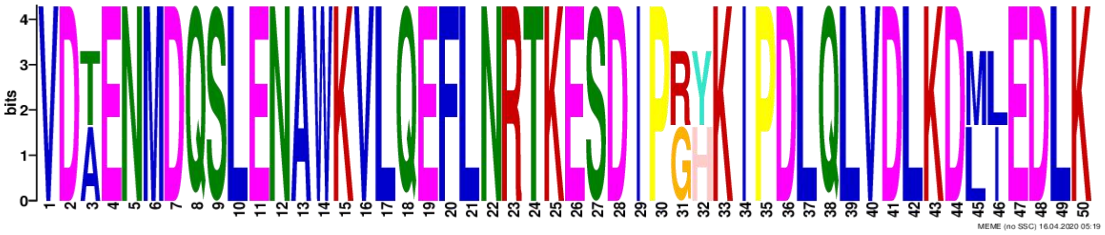

Motif 6

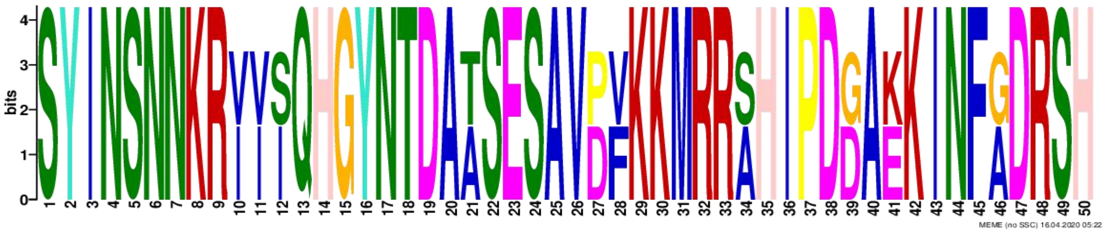

Motif 7

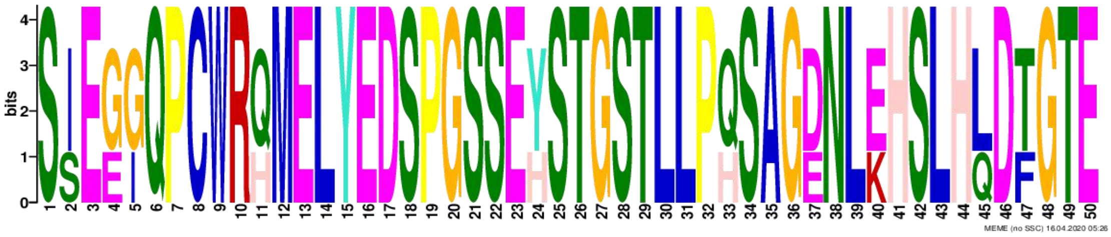

Motif 8

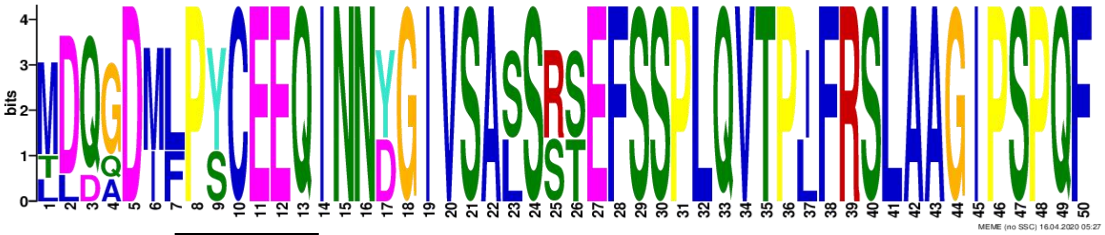

Motif 9

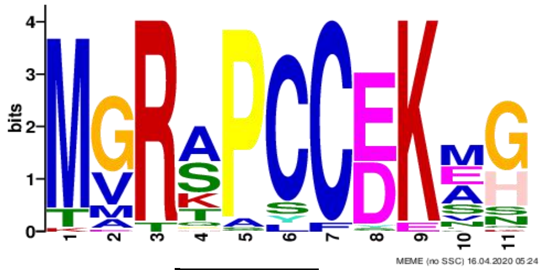

Motif 10

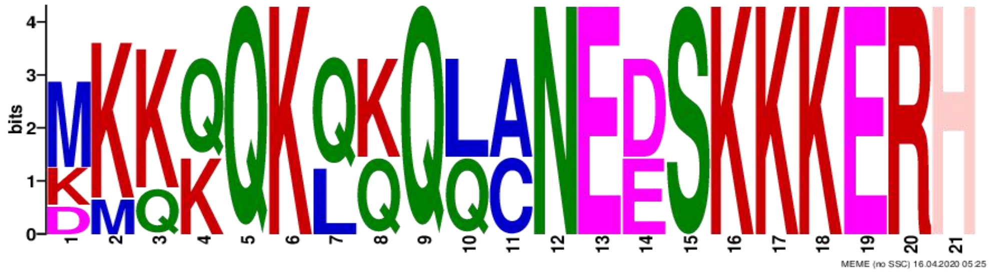

Supplement: Supplementary 6 — ESM_6: logo sequences of each motif of 10 conserved motifs shared by proteins of R2R3-MYB subfamilies. [file 9939403.f6.pdf]
