# Supplementary material for: Identification of Isoflavonoid Biosynthesis-Related R2R3-MYB Transcription Factors in Callerya speciosa (Champ. ex Benth.) Schot Using Transcriptome-Based Gene Coexpression Analysis
Source: Int J Genomics. 2021 May 25;2021:9939403. doi: 10.1155/2021/9939403 (PMC8174187; doi:10.1155/2021/9939403)
Supplement: Supplementary 7 — ESM_7: summary of the BGISEQ-500 RNA-Seq data analysis. [file 9939403.f7.pdf]

ESM_7 Summary of the BGISEQ-500 RNA-Seq data analysis.

Sample	Total Raw Reads (Mb)	Total Clean Reads(Mb)	Clean Reads Ratio (%)	Clean Reads Q20(%)	Clean Reads Q30(%)	Unigene total number	Unigene total length （Mb）	Unigene mean length (bp)	N50	N70	N90	GC percentage (%)	Gene Mapped percentage （%）
6-1	66.24	64.84	97.88	97.23	89.06	55525	62.44	1124	1751	1187	514	42.34	88.40
6-1	66.11	64.72	97.90	97.63	90.28	56129	61.98	1104	1726	1153	498	42.38	88.15
6-3	66.33	64.85	97.76	97.22	89.10	55547	62.17	1119	1731	1171	514	42.34	88.14
12-1	66.38	64.81	97.64	97.09	88.72	57821	66.19	1144	1772	1189	531	41.98	87.27
12-2	66.29	65.10	98.20	97.12	88.63	57717	66.72	1155	1784	1203	542	41.94	87.83
12-3	66.25	64.92	97.99	97.13	88.80	57944	66.66	1150	1778	1196	536	41.93	87.68
18-1	66.18	64.73	97.8	97.22	89.06	61154	63.98	1046	1664	1099	450	42.75	88.18
18-2	68.57	66.94	97.61	97.40	89.58	57913	58.98	1018	1629	1055	435	42.80	87.84
18-3	66.31	64.93	97.92	97.31	89.39	60939	63.68	1045	1661	1089	452	42.69	87.96
30-1	66.34	64.89	97.83	97.06	88.63	68543	76.11	1110	1770	1166	495	41.92	88.22
30-2	68.6	66.83	97.42	97.41	89.56	67216	75.44	1122	1770	1179	506	41.97	88.82
30-3	66.34	65.01	98	97.13	88.82	67465	74.69	1107	1756	1164	493	41.97	88.63
All-Unigene	--	--	--		--	153153	217.31	1418	2167	1497	742	41.67	
Average	66.66	65.21	97.83	97.25	89.14	--	--	--	--			--	88.09
Summary	799.94	782.57	1173.95		--	--	--	--	--			--	--
