# Supplementary material for: Identification of Isoflavonoid Biosynthesis-Related R2R3-MYB Transcription Factors in Callerya speciosa (Champ. ex Benth.) Schot Using Transcriptome-Based Gene Coexpression Analysis
Source: Int J Genomics. 2021 May 25;2021:9939403. doi: 10.1155/2021/9939403 (PMC8174187; doi:10.1155/2021/9939403)
Supplement: Supplementary 8 — ESM_8: sequences of primers designed for RT-qPCR analysis. [file 9939403.f8.pdf]

ESM 8 Sequences of primers designed for RT-qPCR analysis.

Type	Nomenclature used for this paper	Sequence of primers
R2R3-MYB genes	*CsMYB17-F*	GACGAGCGCCTCATAAACT
	CsMYB17-R	ACTCTTGCCACATCTCAGCA
	CsMYB36-F	CCACGGTTGATGAGCTTGC
	CsMYB36-R	CTGATTGCTGGACGGTTGC
	CsMYB41-F	TGATGTTGTGGTTGACGCTG
	CsMYB41-R	GTGCTTCACTGCCCTTGCT
	CsMYB44-F	CTTTGGTCCACGCACCTTTG
	CsMYB44-R	GAGCAGCACAGAGAGTCGTT
	CsMYB45-F	GAGGCTGATGGGGAGGACTA
	CsMYB45-R	GGCTGCCACCACAGTATCTT
	CsMYB46-F	ACCCAAACTGCAAACGAAGC
	CsMYB46-R	ACCATTAGTCCTCCCCGTCA
	CsMYB72-F	TGTTGAGCACTCCATTGGCT
	CsMYB72-R	AGAATCTCCCCACCCTGTGA
	CsMYB81-F	TTCGTTCATGGTCCCGGATG
	CsMYB81-R	CGGTGGTCACTCATAGCTGG
Reference gene	*GAPDH-F*	TTTGGAAGAATCGGGCGTTTGG
	GAPDH-R	TCGGTGGAAATGAAAGGGTCGTTA
Key structural genes	*CsHID-1-F*	TCATCTCAGAAAACCCCTCCA
	CsHID-1-R	CCGCCGTGATAGTAGACCAA
	CsCHI3-F	TCGGGGTTTATCTGGACCCT
	CsCHI3-R	CCAGCTCCTTGGCAGGTTTA
	CsCHS7-F	CACTAAACTTCTGGGTCTTCGC
	CsCHS7-R	GGGCACCTTTGTTGTTCTCG
	CsIFS-F	CTCGACGGCTACGTGATC
	CsIFS-R	CAGAACCAAACGGCAGAA
